# Supplementary material for: Factors associated with protection from MASLD in type 2 diabetes: A prospective study integrating longitudinal MRI/MRE and stable isotope tracing
Source: JHEP Rep. 2026 Jan 8;8(3):101733. doi: 10.1016/j.jhepr.2026.101733 (PMC12914784; doi:10.1016/j.jhepr.2026.101733)

SUPPLEMENTARY MATERIAL

Factors associated with protection from MASLD in type 2 diabetes: A prospective study integrating longitudinal MRI/MRE and stable isotope tracing

Federica Tavaglione[1], Veeral Ajmera[1], Luis Antonio Díaz[1,2], Kelvin Li[3], Egbert Madamba[1], Ricki Bettencourt[1], Lisa Richards[1], Marc Hellerstein[3], Rohit Loomba[1,4]

1 MASLD Research Center, Division of Gastroenterology and Hepatology, University of California at San Diego, La Jolla, California, United States.
2 Departamento de Gastroenterología, Escuela de Medicina, Pontificia Universidad Católica de Chile, Santiago, Chile.
3 Department of Nutritional Sciences & Toxicology, University of California Berkeley, Berkeley, California, United States.
4 School of Public Health, University of California at San Diego, La Jolla, California, United States.

Table of Contents

SUPPLEMENTARY TABLE 1: FACTORS ASSOCIATED WITH PROTECTION FROM MASLD IN INDIVIDUALS WITH TYPE 2 DIABETES. 3

SUPPLEMENTARY TABLE 2: FACTORS ASSOCIATED WITH PROTECTION FROM MASLD IN INDIVIDUALS WITH TYPE 2 DIABETES, CONSIDERING *PNPLA3* UNDER A RECESSIVE MODEL. 4

SUPPLEMENTARY TABLE 3: FACTORS ASSOCIATED WITH PROTECTION FROM MASLD IN INDIVIDUALS WITH TYPE 2 DIABETES, INCLUDING THE POLYGENIC RISK SCORE (PRS) AS A COVARIATE. 5

SUPPLEMENTARY TABLE 4: FACTORS ASSOCIATED WITH PROTECTION FROM MASLD IN INDIVIDUALS WITH TYPE 2 DIABETES, INCLUDING STATIN THERAPY AND TYPE 2 DIABETES DURATION AS COVARIATES. 6

SUPPLEMENTARY FIGURE 1: STUDY FLOWCHART. 7

SUPPLEMENTARY FIGURE 2: COMPARISON OF ORIGINAL AND IMPUTED DISTRIBUTIONS USING DENSITY PLOTS FOR CONTINUOUS VARIABLES AND BAR PLOTS FOR THE CATEGORICAL VARIABLES 8

SUPPLEMENTARY FIGURE 3: FACTORS ASSOCIATED WITH PROTECTION FROM MASLD IN INDIVIDUALS WITH TYPE 2 DIABETES USING REGULARIZED LOGISTIC REGRESSION MODELS. 9

SUPPLEMENTARY FIGURE 4: OVERLAP OF PREDICTORS SELECTED BY BIAS-REDUCED AND PENALIZED LOGISTIC REGRESSION MODELS. 10

Supplementary Table 1: Factors associated with protection from MASLD in individuals with type 2 diabetes.

	OR (95%CI)	p-value
Age (years)	0.98 (0.90, 1.06)	0.594
Sex (Female)	1.21 (0.35, 4.39)	0.758
BMI (kg/m^2)	0.81 (0.64, 0.98)	**0.029**
Waist circumference (cm)	1.04 (0.98, 1.11)	0.178
Hypertension	1.41 (0.51, 4.13)	0.509
Glucose (mg/dL)	1.01 (0.99, 1.03)	0.274
HbA1c (%)	1.13 (0.51, 2.56)	0.757
HOMA-IR	0.59 (0.39, 0.83)	**8.6e-4**
HDL cholesterol (mg/dL)	1.04 (0.99, 1.08)	0.103
Triglycerides (mg/dL)	0.986 (0.973, 0.997)	**0.011**
PNPLA3	0.05 (7.5e-5, 14.40)	0.303
BMI**PNPLA3*	1.09 (0.90, 1.36)	0.380

Outcome: MASLD protection defined as individuals not having steatosis (MRI-PDFF < 5%) and significant fibrosis (MRE < 3 kPa) at both baseline and 2-year follow-up visits.

ORs > 1 indicate increased odds of protection (i.e., higher values of the feature are associated with a higher likelihood of being protected).

ORs < 1 indicate decreased odds of protection (i.e., higher values of the feature are associated with a lower likelihood of being protected).

P values are calculated using Firth logistic regression. Bold values denote statistical significance at p-value <0.05. The *PNPLA3* rs738409 C>G was used as additive model.

Abbreviations: BMI, body mass index; CI, confidence interval; HbA1c, hemoglobin A1c; HOMA-IR, homeostasis model assessment of insulin resistance; OR, odds ratio; *PNPLA3*, patatin-like phospholipase domain-containing protein 3.

Supplementary Table 2: Factors associated with protection from MASLD in individuals with type 2 diabetes, considering *PNPLA3* under a recessive model.

	OR (95%CI)	p-value
Age (years)	0.97 (0.90, 1.04)	0.386
Sex (Female)	1.32 (0.38, 4.90)	0.663
BMI (kg/m^2)	0.83 (0.69, 0.98)	**0.031**
Waist circumference (cm)	1.04 (0.99, 1.11)	0.141
Hypertension	1.40 (0.49, 4.14)	0.530
Glucose (mg/dL)	1.01 (0.99, 1.04)	0.199
HbA1c (%)	0.99 (0.45, 2.21)	0.971
HOMA-IR	0.58 (0.38, 0.83)	**1.10e-3**
HDL cholesterol (mg/dL)	1.03 (0.99, 1.08)	0.189
Triglycerides (mg/dL)	0.986 (0.973, 0.997)	**0.008**
PNPLA3	0.017 (1.21e-21, 4.79e7)	0.629
BMI**PNPLA3*	1.08 (0.44, 4.22)	0.798

Outcome: MASLD protection defined as individuals not having steatosis (MRI-PDFF < 5%) and significant fibrosis (MRE < 3 kPa) at both baseline and 2-year follow-up visits.

ORs > 1 indicate increased odds of protection (i.e., higher values of the feature are associated with a higher likelihood of being protected).

ORs < 1 indicate decreased odds of protection (i.e., higher values of the feature are associated with a lower likelihood of being protected).

P values are calculated using Firth logistic regression. Bold values denote statistical significance at p-value <0.05. The *PNPLA3* rs738409 C>G was used as recessive model.

Abbreviations: BMI, body mass index; CI, confidence interval; HbA1c, hemoglobin A1c; HOMA-IR, homeostasis model assessment of insulin resistance; OR, odds ratio; *PNPLA3*, patatin-like phospholipase domain-containing protein 3.

Supplementary Table 3: Factors associated with protection from MASLD in individuals with type 2 diabetes, including the polygenic risk score (PRS) as a covariate.

	**OR (95%CI)**	**p-value**
Age (years)	0.997 (0.92, 1.09)	0.945
Sex (Female)	1.85 (0.44, 8.68)	0.405
BMI (kg/m^2)	0.82 (0.65, 0.999)	0.**048**
Waist circumference (cm)	1.06 (1.00, 1.14)	0.064
Hypertension	1.53 (0.48, 5.22)	0.469
Glucose (mg/dL)	1.02 (1.00, 1.05)	0.070
HbA1c (%)	0.87 (0.36, 2.12)	0.764
HOMA-IR	0.65 (0.43, 0.89)	**0.007**
HDL cholesterol (mg/dL)	1.01 (0.96, 1.06)	0.649
Triglycerides (mg/dL)	0.986 (0.969, 0.999)	**0.027**
PRS	1.01 (0.03, 45.99)	0.997
BMI*PRS	1.00 (0.88, 1.14)	0.944

Missing values for genetic variants were excluded from the analysis (n = 113).

Outcome: MASLD protection defined as individuals not having steatosis (MRI-PDFF < 5%) and significant fibrosis (MRE < 3 kPa) at both baseline and 2-year follow-up visits.

ORs > 1 indicate increased odds of protection (i.e., higher values of the feature are associated with a higher likelihood of being protected).

ORs < 1 indicate decreased odds of protection (i.e., higher values of the feature are associated with a lower likelihood of being protected).

P values are calculated using Firth logistic regression. Bold values denote statistical significance at p-value <0.05.

Unweighted PRS was calculated as the sum of established risk alleles in *PNPLA3*, *TM6SF2* and *SERPINA1* minus the protective variant in *HSD17B13*.

Abbreviations: BMI, body mass index; CI, confidence interval; HbA1c, hemoglobin A1c; HOMA-IR, homeostasis model assessment of insulin resistance; OR, odds ratio; PRS, polygenic risk score.

Supplementary Table 4: Factors associated with protection from MASLD in individuals with type 2 diabetes, including statin therapy and type 2 diabetes duration as covariates.

	**OR (95%CI)**	**p-value**	**OR (95%CI)***	**p-value**
Age (years)	0.98 (0.91, 1.06)	0.637	0.98 (0.90, 1.08)	0.731
Sex (Female)	1.27 (0.37, 4.56)	0.709	1.83 (0.37, 11.04)	0.466
Diabetes duration (years)	-		1.08 (0.98, 1.20)	0.111
Statins (yes)	0.72 (0.24, 2.14)	0.545	0.70 (0.18, 2.85)	0.610
BMI (kg/m^2)	0.81 (0.65, 0.99)	**0.035**	0.87 (0.69, 1.07)	0.200
Waist circumference (cm)	1.04 (0.98, 1.10)	0.210	1.03 (0.95, 1.11)	0.488
Hypertension	1.37 (0.48, 4.01)	0.553	1.38 (0.40, 5.10)	0.609
Glucose (mg/dL)	1.01 (0.99, 1.03)	0.325	1.02 (0.99, 1.05)	0.215
HbA1c (%)	1.14 (0.51, 2.58)	0.750	0.74 (0.24, 2.17)	0.592
HOMA-IR	0.59 (0.39, 0.83)	**0.001**	0.63 (0.38, 0.90)	**0.007**
HDL cholesterol (mg/dL)	1.03 (0.99, 1.08)	0.119	1.04 (0.99, 1.09)	0.169
Triglycerides (mg/dL)	0.986 (0.973, 0.997)	**0.010**	0.985 (0.968, 0.998)	**0.024**
PNPLA3	0.06 (8.88e-05, 16.01)	0.324	0.02 (7.07e-06, 10.06)	0.220
BMI**PNPLA3*	1.09 (0.89, 1.36)	0.401	1.13 (0.91, 1.47)	0.273

* Missing values for type 2 diabetes duration were excluded from the analysis (n = 115).

Outcome: MASLD protection defined as individuals not having steatosis (MRI-PDFF < 5%) and significant fibrosis (MRE < 3 kPa) at both baseline and 2-year follow-up visits.

ORs > 1 indicate increased odds of protection (i.e., higher values of the feature are associated with a higher likelihood of being protected).

ORs < 1 indicate decreased odds of protection (i.e., higher values of the feature are associated with a lower likelihood of being protected).

P values are calculated using Firth logistic regression. Bold values denote statistical significance at p-value <0.05. The *PNPLA3* rs738409 C>G was used as additive model.

Abbreviations: BMI, body mass index; CI, confidence interval; HbA1c, hemoglobin A1c; HOMA-IR, homeostasis model assessment of insulin resistance; OR, odds ratio; *PNPLA3*, patatin-like phospholipase domain-containing protein 3.

Supplementary Figure 1: Study flowchart.

DOS study

N = 622 (MARCH 2025)	
**Inclusion criteria**<br>• Age ≥ 50 years and <80 years<br>• Type 2 diabetes according to the ADA clinical practice recommendations<br>• Written informed consent	
**Baseline**	622
**2-year follow-up**	204

Exclusion criteria		
	**Baseline**	**2-year follow-up**
**MRI-PDFF NA**	56	34
**MRE NA**	95	37
**Alcohol >20/30 g/day (F/M)**	20	-
**Alcohol g/day NA**	32	-

n = 148	
**Inclusion criteria**<br>• Paired MRI and MRE at baseline and 2-year follow-up	
**Baseline**	148
**2-year follow-up**	148

Supplementary Figure 2: Comparison of original and imputed distributions using density plots for continuous variables and bar plots for the categorical variables. *Abbreviations*: HbA1c, hemoglobin A1c; HDL, high-density lipoprotein; HOMA-IR, homeostasis model assessment of insulin resistance; *PNPLA3*, patatin-like phospholipase domain-containing protein 3.

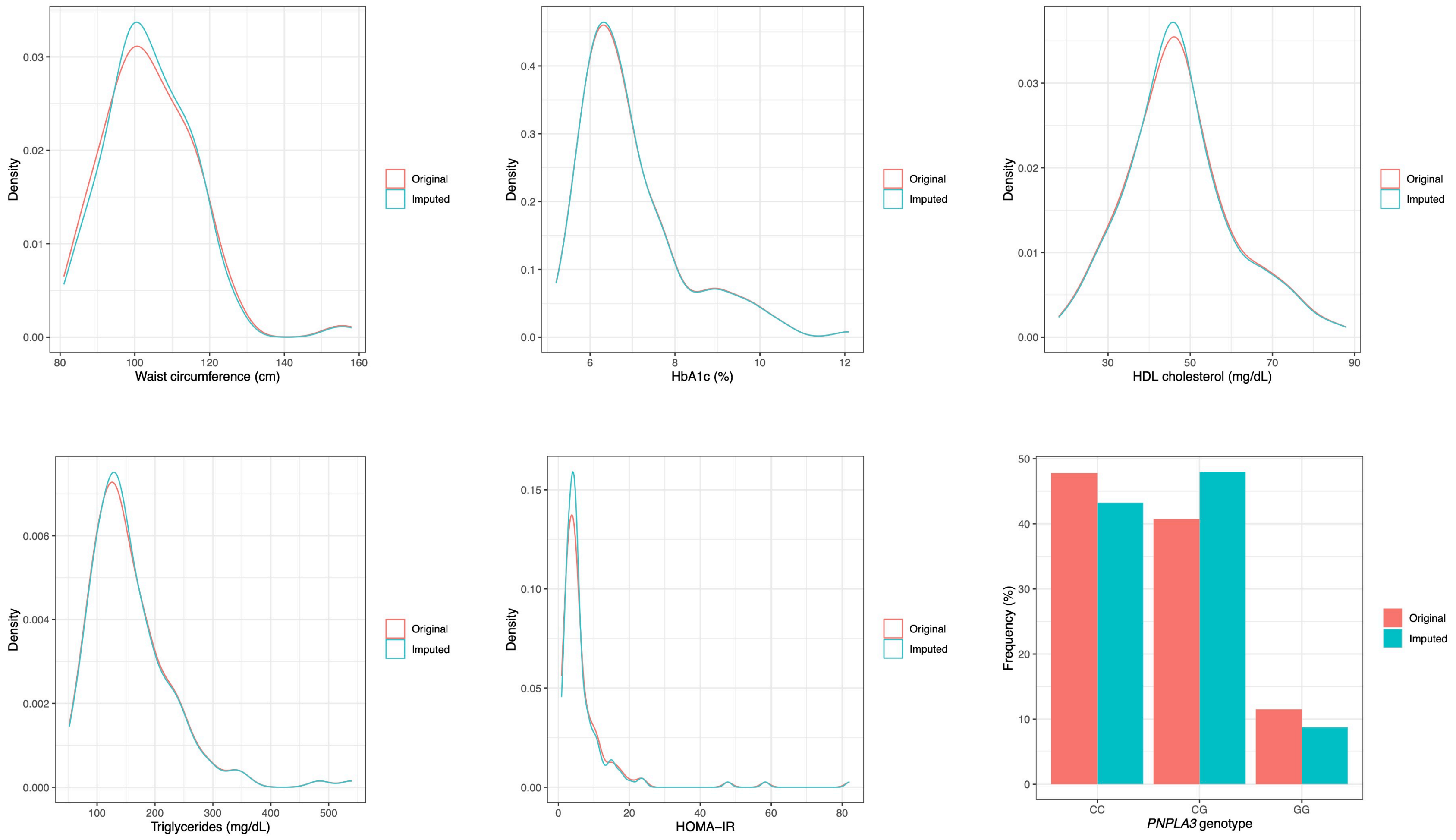

Supplementary Figure 3: Factors associated with protection from MASLD in individuals with type 2 diabetes using regularized logistic regression models. All continuous predictors were standardized (z-scores) prior to inclusion in the regularized regression models to ensure appropriate penalization and variable selection. *Abbreviations*: BMI, body mass index; HbA1c, hemoglobin A1c; HDL-C, high-density lipoprotein cholesterol; HOMA-IR, homeostasis model assessment of insulin resistance; *PNPLA3*, patatin-like phospholipase domain-containing protein 3.

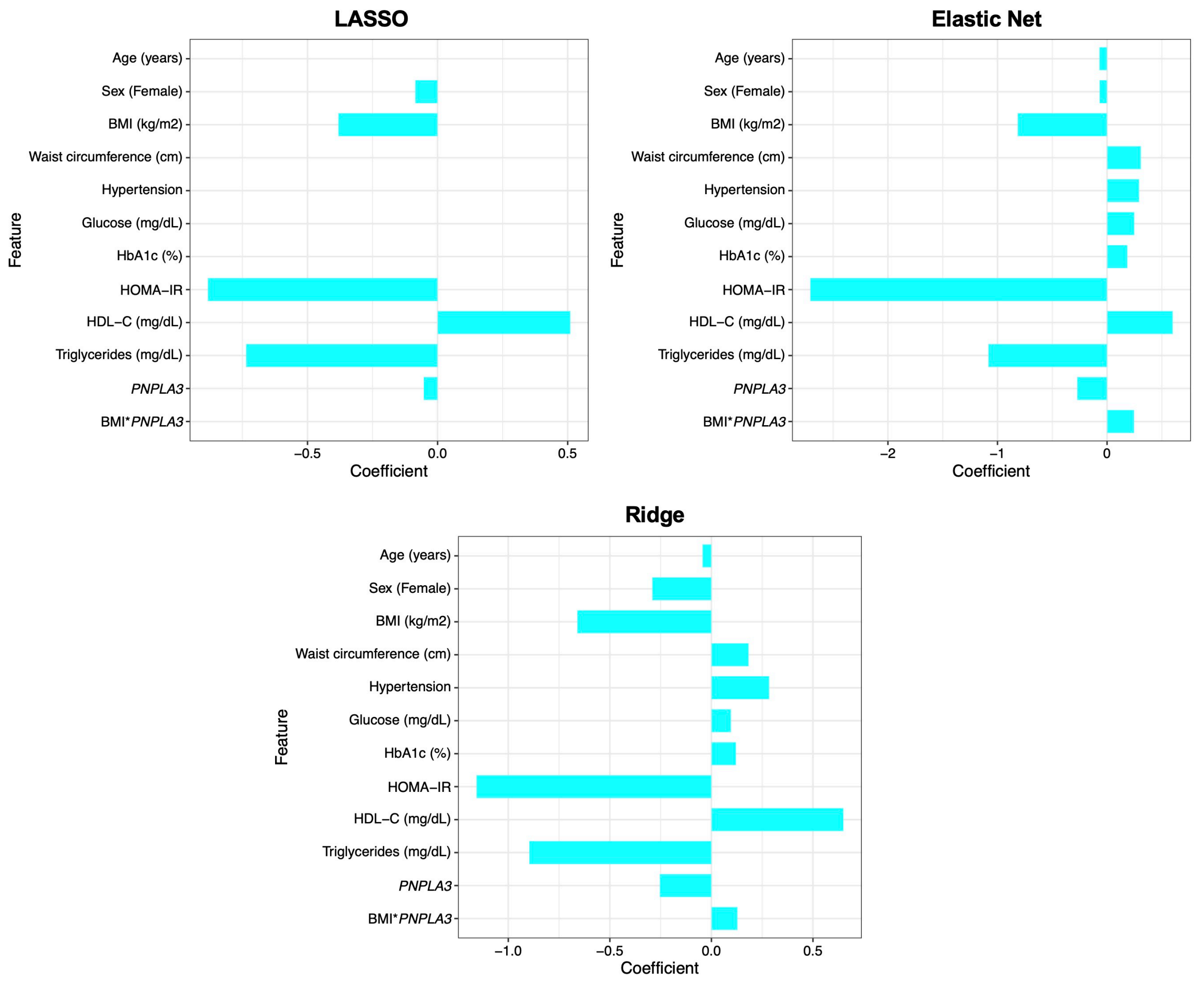

Supplementary Figure 4: Overlap of predictors selected by bias-reduced and penalized logistic regression models. The Euler diagram shows the overlap of predictors retained by Firth logistic regression, LASSO, Ridge, and Elastic Net models. Each method applies different regularization or bias reduction strategies to address multicollinearity and overfitting. Common predictors across models suggest robust associations with the outcome. Predictors consistently selected by all models are displayed in bold. *Abbreviations*: BMI, body mass index; HbA1c, hemoglobin A1c; HDL-C, high-density lipoprotein cholesterol; HOMA-IR, homeostasis model assessment of insulin resistance; *PNPLA3*, patatin-like phospholipase domain-containing protein 3.

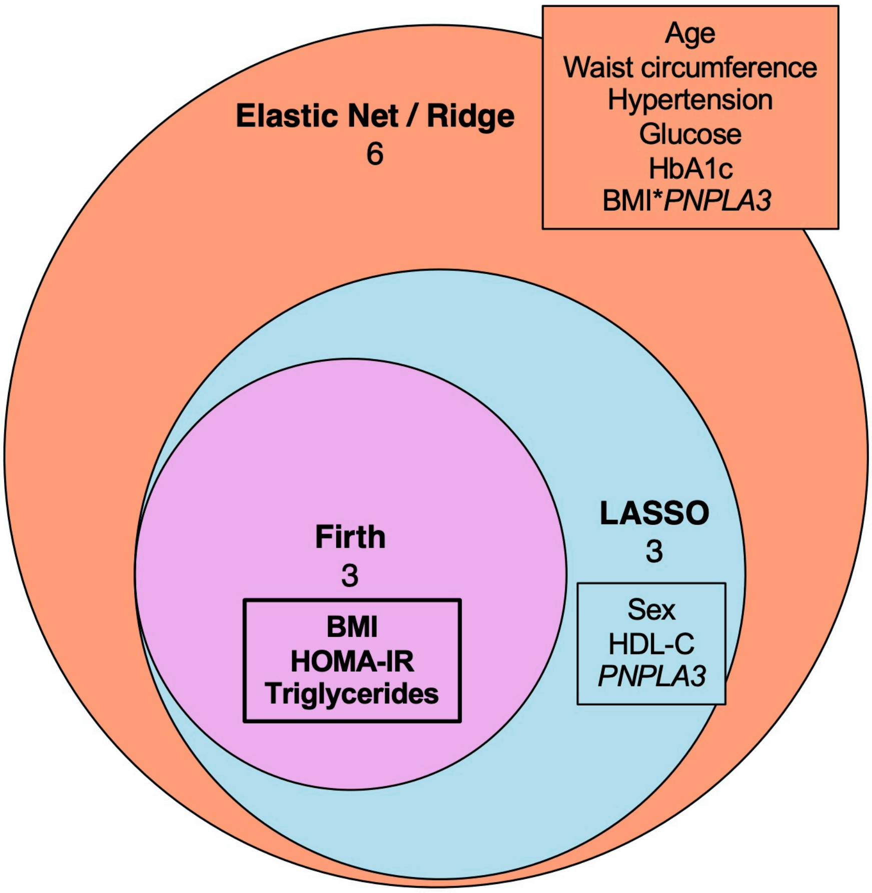

Supplement: Multimedia component 1 [file mmc1.pdf]
