# Supplementary material for: Factors associated with protection from MASLD in type 2 diabetes: A prospective study integrating longitudinal MRI/MRE and stable isotope tracing
Source: JHEP Rep. 2026 Jan 8;8(3):101733. doi: 10.1016/j.jhepr.2026.101733 (PMC12914784; doi:10.1016/j.jhepr.2026.101733)
Supplement: Multimedia component 2 [file mmc2.docx]

**JHEP Reports**

**CTAT methods**

Tables for a “Complete, Transparent, Accurate and Timely account” (CTAT) are now mandatory for all revised submissions. The aim is to enhance the reproducibility of methods.

- Only include the parts relevant to your study
- Refer to the CTAT in the main text as ‘Supplementary CTAT Table’
- Do not add subheadings
- Add as many rows as needed to include all information
- Only include one item per row

**If the CTAT form is not relevant to your study, please outline the reasons why:**

|  |
| --- |

- 1. **Antibodies**

| **Name** | **Citation** | **Supplier** | **Cat no.** | **Clone no.** |
| --- | --- | --- | --- | --- |
| N/A |  |  |  |  |

- 1. **Cell lines**

| **Name** | **Citation** | **Supplier** | **Cat no.** | **Passage no.** | **Authentication test method** |
| --- | --- | --- | --- | --- | --- |
| N/A |  |  |  |  |  |

- 1. **Organisms**

| **Name** | **Citation** | **Supplier** | **Strain** | **Sex** | **Age** | **Overall n number** |
| --- | --- | --- | --- | --- | --- | --- |
| N/A |  |  |  |  |  |  |

- 1. **Sequence based reagents**

| **Name** | **Sequence** | **Supplier** |
| --- | --- | --- |
| N/A |  |  |

- 1. **Biological samples**

| **Description** | **Source** | **Identifier** |
| --- | --- | --- |
| Whole blood | UCSD MASLD Research Center | IRB #160231 |
| Human plasma | UCSD MASLD Research Center | IRB #140338 |

- 1. **Deposited data**

| **Name of repository** | **Identifier** | **Link** |
| --- | --- | --- |
| N/A |  |  |

- 1. **Software**

| **Software name** | **Manufacturer** | **Version** |
| --- | --- | --- |
| R statistical software | R Foundation for Statistical Computing, Vienna, Austria | 4.4.2 |

- 1. **Other (*e.g*. drugs, proteins, vectors etc.)**

| N/A |  |  |
| --- | --- | --- |

- 1. **Please provide the details of the corresponding methods author for the manuscript:**

| - Federica Tavaglione, MD, PhD, MASLD Research Center, Division of Gastroenterology and Hepatology, University of California at San Diego, Altman Clinical and Translational Research Institute, 9500 Gilman Drive La Jolla, CA, 92093‐0887. Email: [ftavaglione@health.ucsd.edu](mailto:ftavaglione@health.ucsd.edu). - Rohit Loomba, MD, MHSc, MASLD Research Center, Division of Gastroenterology and Hepatology, University of California at San Diego, Altman Clinical and Translational Research Institute, 9500 Gilman Drive La Jolla, CA, 92093‐0887. Email: [roloomba@health.ucsd.edu](mailto:roloomba@health.ucsd.edu). |
| --- |

**2.0 Please confirm for randomised controlled trials all versions of the clinical protocol are included in the submission. These will be published online as supplementary information.**

|  |
| --- |
