# Supplementary material for: Factors associated with protection from MASLD in type 2 diabetes: A prospective study integrating longitudinal MRI/MRE and stable isotope tracing
Source: JHEP Rep. 2026 Jan 8;8(3):101733. doi: 10.1016/j.jhepr.2026.101733 (PMC12914784; doi:10.1016/j.jhepr.2026.101733)

Research article

JHEP Reports

Factors associated with protection from MASLD in type 2 diabetes: A prospective study integrating longitudinal MRI/MRE and stable isotope tracing

Authors

Federica Tavaglione, Veeral Ajmera, Luis Antonio Díaz, ..., Lisa Richards, Marc Hellerstein, Rohit Loomba

Correspondence

roloomba@health.ucsd.edu (R. Loomba), ftavaglione@health.ucsd.edu (F. Tavaglione).

Graphical abstract

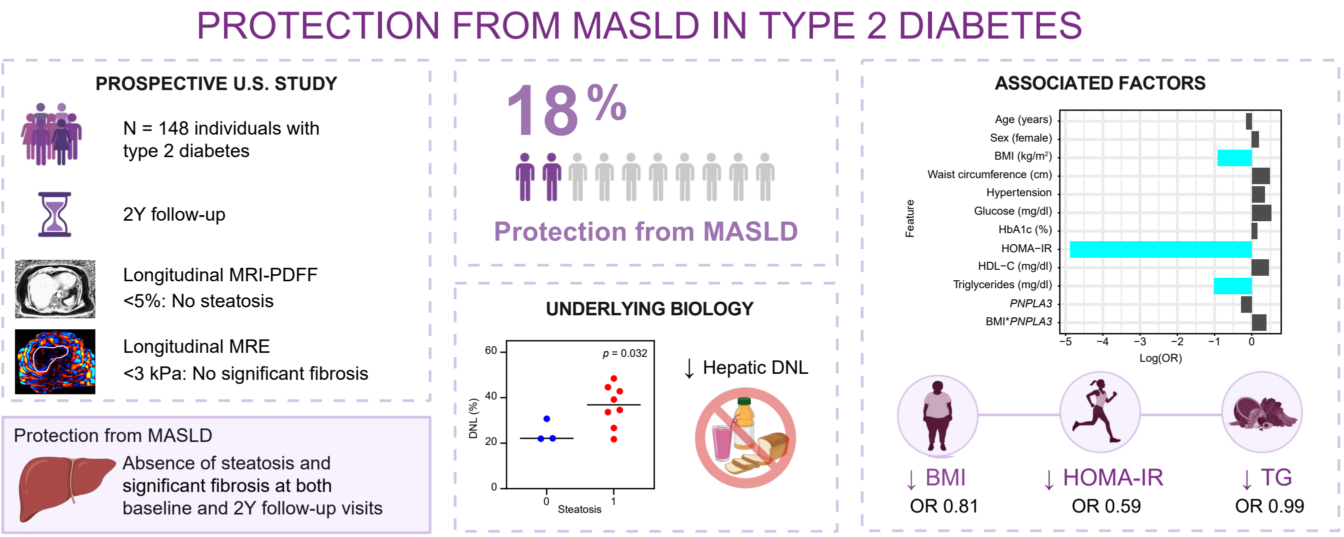

Highlights:

- Some individuals with type 2 diabetes appear protected from MASLD, but the factors driving this protection remain unclear.
- In a 2-year prospective type 2 diabetes cohort, lower BMI, lower HOMA-IR, and lower triglycerides predicted MRI-defined protection from MASLD.
- Reduced hepatic *de novo* lipogenesis may underlie this protected phenotype.
- These findings could guide strategies to lower MASLD risk and progression in patients with type 2 diabetes.

Impact and implications:

Understanding factors conferring protection from metabolic dysfunction-associated steatotic liver disease (MASLD) in individuals with type 2 diabetes represents a novel research avenue which has not been systematically explored. In this prospective cohort of 148 individuals with type 2 diabetes undergoing advanced liver phenotyping at baseline and 2-year follow-up, we identified lower BMI, lower HOMA-IR, and lower circulating triglycerides as the strongest predictors of remaining free of MASLD. Additionally, by performing an exploratory analysis in an independent cohort of 11 individuals with type 2 diabetes with tracer data, we found that lower hepatic *de novo* lipogenesis may underlie this protective phenotype. These findings suggest that managing modifiable risk factors, such as body weight and lipid profile, may be critical for preventing MASLD development and progression in individuals with type 2 diabetes.

https://doi.org/10.1016/j.jhepr.2026.101733
© 2026 The Author(s). Published by Elsevier B.V. on behalf of European Association for the Study of the Liver (EASL). This is an open access article under the CC BY license (http://creativecommons.org/licenses/by/4.0/). JHEP Reports, 2026, 8, 1–9

Factors associated with protection from MASLD in type 2 diabetes: A prospective study integrating longitudinal MRI/MRE and stable isotope tracing

Federica Tavaglione[1,*], Veeral Ajmera[1], Luis Antonio Díaz[1,2], Kelvin Li[3], Egbert Madamba[1], Ricki Bettencourt[1], Lisa Richards[1], Marc Hellerstein[3], Rohit Loomba[1,4,*]

JHEP Reports **2026**. vol. 8 | 1–9

Check for updates

Background & Aims: Type 2 diabetes is one of the strongest risk factors for the development and progression of metabolic dysfunction-associated steatotic liver disease (MASLD). In this study, we aimed to identify factors associated with protection from MASLD in a prospective cohort of individuals with type 2 diabetes.

Methods: This prospective study included 148 individuals with type 2 diabetes who underwent advanced liver phenotyping using MRI and MRE techniques at baseline and 2-year follow-up. Protection from MASLD was defined as the absence of hepatic steatosis (MRI-proton density fat fraction <5%) and significant fibrosis (MRE <3 kPa) at both time points. Factors associated with protection from MASLD were assessed using Firth's penalized logistic regression. Regularized logistic regression models were fitted as complementary analyses.

Results: The mean (SD) age and BMI were 65 (8) years and 30.4 (4.3) kg/m^2, respectively. After a median follow-up of 2 (1.5–2.4) years, 27 (18%) individuals demonstrated an absence of MASLD. Across all modeling approaches, lower BMI (odds ratio [OR] 0.81, 95% CI 0.64–0.98; p = 0.029), lower HOMA-IR (OR 0.59, 95% CI 0.39–0.83; p = 8.6e-4), and lower circulating triglycerides (OR 0.986, 95% CI 0.973–0.997; p = 0.011) emerged as the strongest predictors of protection from MASLD. An exploratory analysis of 11 individuals with type 2 diabetes from an independent cohort, with hepatic *de novo* lipogenesis quantified by stable isotope tracing, revealed lower hepatic *de novo* lipogenesis in those free from MASLD.

Conclusions: Managing modifiable risk factors, such as body weight and lipid profile, may be critical for preventing MASLD development in individuals with type 2 diabetes.

© 2026 The Author(s). Published by Elsevier B.V. on behalf of European Association for the Study of the Liver (EASL). This is an open access article under the CC BY license (http://creativecommons.org/licenses/by/4.0/).

Introduction

Paralleling the global burden of obesity and type 2 diabetes, metabolic dysfunction-associated steatotic liver disease (MASLD) is a leading cause of chronic liver disease worldwide, affecting up to 38% of the global adult population. MASLD encompasses a dynamic spectrum of conditions, ranging from isolated lipid accumulation in the liver (hepatic steatosis) to inflammation (steatohepatitis, MASH) and fibrosis, ultimately leading to cirrhosis and hepatocellular carcinoma.[1] The underlying pathophysiology of MASLD is inherently complex and varies among individuals, contributing to differences in disease progression, development of comorbidities, and treatment response.[2–4] Notably, not all individuals diagnosed with MASLD will experience progression to MASH or advanced stages of liver disease. Additionally, MASLD is associated with an increased risk of extrahepatic complications, such as cardiovascular disease and type 2 diabetes, which also varies among individuals.[5,6] This variability in the severity and progression of MASLD, along with its extrahepatic manifestations, underscores its intrinsic heterogeneity and highlights the importance of individualized risk stratification and therapeutic strategies.[2,3]

Over the past decades, research has focused on identifying genetic, metabolic, and environmental risk factors that drive the development of MASLD and shape its natural history. Among these, type 2 diabetes consistently emerges as one of the strongest determinants of MASLD onset and progression.[3,7–9] Approximately two-thirds of individuals with type 2 diabetes have MASLD, and about 1 in 6 show evidence of advanced fibrosis.[10,11] Furthermore, in individuals with MASLD, the presence of type 2 diabetes is associated with more than a two-fold increased risk of hepatic decompensation and over a five-fold increased risk of hepatocellular carcinoma.[12]

Intriguingly, some individuals with type 2 diabetes do not develop MASLD and remain free from liver disease over time. Understanding the factors that confer such protection

* Corresponding authors. Address: University of California at San Diego, Altman Clinical and Translational Research Institute, 9500 Gilman Drive La Jolla, CA, United States. Tel.: +92093-0887.
E-mail addresses: roloomba@health.ucsd.edu (R. Loomba), ftavaglione@health.ucsd.edu (F. Tavaglione).
https://doi.org/10.1016/j.jhepr.2026.101733

represents a novel and impactful research avenue with the potential to inform strategies that reduce liver disease risk and progression in this high-risk population. Yet, this question has not been systematically addressed. In this study, we aimed to identify determinants of protection from MASLD in a prospective cohort of individuals with type 2 diabetes who underwent advanced liver phenotyping using magnetic resonance imaging (MRI) and magnetic resonance elastography (MRE) techniques at baseline and 2-year follow-up. Additionally, we performed an exploratory analysis from an independent study using stable isotope tracing to investigate potential biological mechanisms underlying the non-MASLD status in this population.

Patients and methods

Study cohort

The study design and methods of the "Type 2 Diabetes Observational Study (DOS)" have been described in detail previously.[11] Briefly, consecutive participants aged 50–80 years old with a diagnosis of type 2 diabetes according to the American Diabetes Association clinical practice recommendations were prospectively enrolled into the study (Fig. S1). Participants were excluded if they reported excessive alcohol intake for a period longer than 2 years at any time in the last 10 years or had laboratory evidence of liver disease other than MASLD. Participants were recruited from primary care and endocrinology clinics in the greater San Diego area. Participants were also recruited through the distribution of educational brochures, ads in local newspapers, local fairs, and social media. All participants underwent a standardized research visit at baseline and 2-year follow-up, including medical history, physical examination, laboratory investigation, and advanced liver imaging using MRI-derived proton density fat fraction (MRI-PDFF) and MRE, as well as vibration-controlled transient elastography with controlled attenuation parameter assessment, between 2016 and 2025 at the UCSD MASLD Research Center, La Jolla, California, United States. The DOS study has been approved by the UCSD Institutional Review Board (IRB #160231) and it was conducted in accordance with the principles of the Declaration of Helsinki. All participants gave written informed consent to the study. This work is compliant with the STROBE (Strengthening the Reporting of Observational studies in Epidemiology) guidelines.[13]

Liver imaging

Participants underwent a non-contrast MR exam with liver fat quantification and liver stiffness assessment using MRI-PDFF and MRE, respectively. To date, MRI-PDFF and MRE are the most accurate non-invasive biomarkers for quantifying hepatic steatosis and fibrosis, respectively. Imaging was performed at the UCSD MR3T Research Laboratory using a 3T research scanner (GE Signa EXCITE HDxt; GE Healthcare, Waukesha, WI). Liver stiffness data were obtained using 2D MRE at 60 Hz. Acquired MR images were interpreted by a radiologist who was blinded to clinical and laboratory data.[11]

Outcome measures

Non-MASLD status was defined as absence of steatosis, indicated by MRI-PDFF <5%,[14] and absence of significant fibrosis, indicated by MRE <3 kPa.[11,15] Protection from MASLD was defined as maintenance of a non-MASLD status, *i.e.* absence of steatosis and significant fibrosis, at both baseline and 2-year follow-up time points.

Genotyping

Genomic data at UCSD were extracted from low-coverage whole genome sequencing provided by Color Health©, Inc. This method involves sampling locations across the entire genome at a low depth, followed by imputations that statistically infer missing information that occurs as a result of the sampling, and it is comparable to genotyping arrays.[16] *PNPLA3* rs738409 C>G (p.I148M), *TM6SF2* rs58542926 C>T (p.E167 K), *SERPINA1* rs28929474 C>T (p.E342 K), and *HSD17B13* rs72613567 T>TA were coded as 0, 1, or 2, corresponding to non-carriers, heterozygous carriers and homozygous carriers of the minor allele, respectively. For *PNPLA3* rs738409, *TM6SF2* rs58542926, and *SERPINA1* rs28929474 the minor allele (*i.e.* G allele, T allele, and T allele, respectively) was the risk-increasing allele, whereas for *HSD17B13* rs72613567 the minor TA allele had a protective effect.[17–20]

An unweighted polygenic risk score (PRS) was created as the sum of established risk alleles in *PNPLA3*, *TM6SF2* and *SERPINA1* minus the protective variant in *HSD17B13*.[21]To analyze genetic risk, PRS was dichotomized into high and low groups according to the median.

Determination of fasting hepatic de novo lipogenesis

In this exploratory analysis, we included participants with type 2 diabetes who had available hepatic *de novo* lipogenesis (DNL) measurements from an independent study, the "Development of Kinetic Biomarkers of Liver Fibrosis based on Stable Isotope Mass Spectrometry Techniques for Measuring in Nonalcoholic Fatty Liver Disease (NAFLD)" study. Eligible individuals were adults (≥18 years) with a diagnosis of type 2 diabetes according to the American Diabetes Association clinical practice recommendations, without excessive alcohol consumption and other causes of liver disease. Participants underwent a non-contrast MR exam with liver fat quantification and liver stiffness assessment using MRI-PDFF and MRE, as previously described.[11] Fractional DNL was determined by the quantification of newly synthesized palmitate through the DNL pathway in plasma samples in the fasting state after a 21-day labeling period. Deuterated water (^2H_2O) was administered to patients orally. Patients consumed 50 ml of 70% deuterated water (Cambridge Isotope Laboratories, Tewksbury, MA) 3 times daily for 4 days followed by 50 ml of 70% deuterated water twice a day for a total of 21 days. Fasting plasma samples were drawn for gas chromatography–mass spectroscopy analysis on day 21 after the onset of labeling. Plasma triglyceride-palmitate from the 21-day time point was esterified and analyzed for mass isotopomer abundances by gas chromatography–mass spectroscopy using mass isotopomer distribution analysis to determine the precursor and product enrichments for the calculation of fractional DNL.[22] Analyses of samples for fractional DNL were performed at the University of California, Berkeley. The study has been approved by the UCSD Institutional Review Board (IRB #140338) and it was conducted in accordance with the principles of the Declaration of Helsinki. All participants provided written informed consent.

Statistical analysis

Continuous variables are reported as mean (SD) or median (IQR), as appropriate. Categorical variables are summarized as frequencies (percentages). Clinical, biochemical, and imaging characteristics of the study population, stratified by liver disease status, were compared using ANOVA, the Kruskal–Wallis test, Chi-squared test, or Fisher's exact test as appropriate.

Factors associated with protection from MASLD were assessed using Firth's penalized logistic regression. As complementary analyses, regularized logistic regression models were fitted using least absolute shrinkage and selection operator (LASSO, L1 penalty), ridge (L2 penalty), and elastic net (a combination of L1 and L2 penalties). Variables included in the models were baseline age (years), sex (female), cardiometabolic risk factors (including BMI [kg/m^2], waist circumference [cm], glucose [mg/dl], HbA1c [%], HDL cholesterol [mg/dl], homeostasis model assessment of insulin resistance (HOMA-IR), and triglycerides [mg/dl][23]), the *PNPLA3* rs738409 C>G genotype (additive model), and the BMI × *PNPLA3* interaction term. All continuous predictors were standardized (z-scores) prior to inclusion in the regularized regression models to ensure appropriate penalization and variable selection. Continuous variables were not standardized in the Firth logistic regression model to retain interpretability of the effect estimates in their original units. For the regularized models, 10-fold cross-validation was performed to determine the optimal shrinkage parameter (λ) by minimizing the cross-validated deviance. For the elastic net model, the mixing parameter (α) was set to 0.5 to balance L1 and L2 penalties. Final models were fitted using the selected λ on the full dataset. Predictors with non-zero coefficients in the LASSO, ridge, and elastic net models were considered retained features, and their direction of association was compared with that observed in the primary Firth model to assess consistency. Sensitivity analyses were performed considering *PNPLA3* under a recessive model and including the PRS, type 2 diabetes duration, and statin therapy as covariates.

Missing data were imputed using weighted k-nearest neighbors using the VIM package in R. Imputation quality was visually assessed by comparing the distributions of original and imputed values using density plots for continuous variables and bar plots for the categorical genotype variable, indicating no major distortion due to imputation (Fig. S2). For the categorical variable, a chi-squared test was also performed, confirming that the distributions before and after imputation were comparable (p = 0.467). Multicollinearity was defined as a variance inflation factor greater than 5. All statistical analyses were conducted using R version 4.4.2 (R Foundation for Statistical Computing, Vienna, Austria). Two-sided p values <0.05 were considered statistically significant.

Results

Clinical characteristics of the study cohort

A total of 148 participants with type 2 diabetes from the DOS study, who had paired MRI-PDFF and MRE assessments at baseline and 2-year follow-up, were included in the analyses (Fig. S1). Participant characteristics at the baseline assessment visit, stratified by liver disease status, are presented in Table 1. In the overall cohort, the mean (SD) age and BMI were 65 (8) years and 30.4 (4.3) kg/m^2, respectively. Women comprised 61% of the cohort, and 31% participants were of Hispanic ethnicity. Median (IQR) MRI-PDFF and MRE values were 8.6% (4.2–14.3) and 2.4 kPa (2.1–2.9), respectively. The minor allele frequency of the *PNPLA3* I148M variant was 0.32, and genotype distribution was in Hardy-Weinberg equilibrium.

The natural history of liver disease in the study cohort is illustrated in Fig. 1. After a median follow-up of 2 (1.5–2.4) years, 27 (18%) remained free from liver disease at both baseline and 2-year follow-up and were classified as protected, while 10 (7%) developed liver disease over time and 111 (75%) had sustained liver disease. No participants with baseline liver disease reverted to a non-MASLD status. Protected individuals had lower BMI and waist circumference, lower liver enzymes, and a more favorable lipid profile, including lower circulating triglycerides and higher HDL cholesterol (p <0.05). They were also less frequently treated with glucagon-like peptide-1 receptor agonists (p = 0.044). No significant differences were observed in glucose or HbA1c levels between the two groups. None of the protected individuals were homozygous carriers of the *PNPLA3* risk allele (Table 1). Upon stratification by BMI categories, the majority of protected individuals carrying either the *PNPLA3* CC (wild type) or CG genotype had a BMI <25 kg/m^2 (50% and 33.3%, respectively) (Fig. 2), supporting the well-documented synergistic effect between the *PNPLA3* I148M variant and obesity on MASLD development and progression.

The distribution of protection from MASLD across well-established machine-learning–derived subtypes of type 2 diabetes[24–27] is shown in Fig. 4. These subtypes were assigned following the originally reported sex-stratified pipeline and the nearest-centroid classification approach.[24] Glutamic acid decarboxylase antibody status was assumed negative for all participants. HOMA-IR and homoeostasis model assessment of β-cell function (HOMA-B) were calculated based on fasting glucose and fasting insulin measurements.[28] Notably, individuals classified as having the severe insulin-resistant diabetes (SIRD) subtype exhibited the lowest prevalence of protection from MASLD, highlighting the central role of insulin resistance in liver disease susceptibility.

Factors associated with protection from MASLD development in type 2 diabetes

Firth's penalized logistic regression was employed as the primary modeling approach to identify predictors independently associated with protection from MASLD (Fig. 3, Table S1). This method was chosen to reduce bias in maximum likelihood estimation, particularly given the relatively small number of protected individuals in the cohort. Lower BMI (odds ratio [OR] 0.81; 95% CI 0.64–0.98; p = 0.029), lower HOMA-IR (OR 0.59; 95% CI 0.39–0.83; p = 8.6e-4), and lower circulating triglycerides (OR 0.986; 95% CI 0.973–0.997; p = 0.011) emerged as the strongest predictors of protection from MASLD (Table S1).

To confirm the robustness of the findings, we conducted complementary analyses using regularized logistic regression models, including LASSO, ridge, and elastic net (Fig. S3). These techniques help address potential multicollinearity and overfitting by applying different forms of penalization. Across all modeling approaches, lower BMI, lower HOMA-IR, and

Table 1. Clinical characteristics of the overall cohort at the baseline assessment visit, stratified by protection from MASLD development.

	Overall	MASLD	Non-MASLD	*p* value
N (%)	148 (100%)	121 (82%)	27 (18%)	-
Follow-up, years	2 (1.5-2.4)	2 (1.5-2.4)	2 (1.6-2.3)	0.817
**Clinical data**				
Age, years	65 (8)	65 (7)	66 (9)	0.460
Age ≥65 years, n (%)	83 (56%)	67 (55%)	16 (59%)	0.878
Women, n (%)	91 (61%)	75 (62%)	16 (59%)	0.965
BMI, kg/m^2	30.4 (4.3)	30.9 (4.2)	28.2 (4.2)	**0.003**
BMI categories, n (%)				**0.010**
<25 kg/m^2	20 (14%)	12 (10%)	8 (30%)	
25-30 kg/m^2	46 (31%)	36 (30%)	10 (37%)	
≥30 kg/m^2	82 (55%)	73 (60%);	9 (33%)	
BMI absolute change from baseline to follow-up, kg/m^2	-0.3 (-1.5-0.9)	-0.2 (-1.6-0.9)	-0.3 (-1.1-1)	0.598
Waist circumference, cm	103 (96-112)	104 (96-113)	98 (91-105)	**0.049**
Systolic blood pressure, mmHg	130 (120-141)	131 (120-140)	129 (118-148)	0.739
Diastolic blood pressure, mmHg	72 (66-80)	73 (67-80)	70 (62-80)	0.467
Ethnicity/race, n (%)				0.587
American Indian	2 (1%)	2 (2%)	0 (0%)	
Asian	25 (17%)	21 (17%)	4 (15%)	
Black	4 (3%)	3 (2%)	1 (4%)	
White	68 (46%)	55 (45%)	13 (48%)	
Hispanic	46 (31%)	38 (31%)	8 (30%)	
Pacific Islands	2 (1%)	2 (2%)	0 (0%)	
Other	1 (1%)	0 (0%)	1 (4%)	
Diabetes duration, years	7 (3-14)	5 (3-14)	10 (8-17)	**0.021**
NA	33	25	8	
Hypertension, n (%)	87 (59%)	72 (60%)	15 (56%)	0.872
**Lifestyle**				
Alcohol, g/day	0 (0-2.8)	0 (0-2.8)	0.9 (0-2.8)	0.558
Moderate-vigorous physical activity, yes	56 (41%)	47 (41%)	9 (41%)	1
**Biochemistry**				
Glucose, mg/dl	123 (102.8-153)	126 (104-156)	110 (99.5-139.5)	0.141
HbA1c, %	6.6 (6.1-7.5)	6.7 (6.2-7.6)	6.4 (5.8-7)	0.063
HOMA-IR	4.6 (3-8.1)	5.1 (3.6-8.8)	2.7 (1.9-4)	**<0.001**
Cholesterol, mg/dl	167.5 (41.1)	168.9 (42.4)	161.1 (34.5)	0.381
HDL cholesterol, mg/dl	46 (38-54)	45 (38-51)	58.5 (45.5-66)	**<0.001**
LDL cholesterol, mg/dl	84 (64.8-107)	85 (65-109.5)	73 (62.5-94.8)	0.113
Triglycerides, mg/dl	139 (110-186)	153 (113.5-199.5)	115 (85.2-142)	**<0.001**
ALT, U/L	28 (20-43)	31 (22-45)	18 (13-22)	**<0.001**
AST, U/L	26 (19-35)	26 (21-38)	19 (14-26)	**<0.001**
GGT, U/L	30 (21-51)	33 (24-53)	19 (16-33)	**<0.001**
ALP, U/L	74 (60-94)	75 (59-95)	72 (64-83)	0.844
Bilirubin, mg/dl	0.5 (0.3-0.6)	0.4 (0.3-0.6)	0.5 (0.3-0.7)	0.515
Albumin, g/dl	4.5 (4.3-4.6)	4.5 (4.3-4.6)	4.4 (4.2-4.7)	0.233
Platelets, cells/µl	256 (71)	257 (71)	249 (74)	0.597
**Liver imaging**				
MRI-PDFF, %	8.6 (4.2-14.3)	10.8 (6.7-15.7)	2.4 (1.5-3.3)	**<0.001**
MRE, kPa	2.4 (2.1-2.9)	2.4 (2.1-3)	2.3 (2-2.4)	**0.003**
Steatosis, n (%)	103 (70%)	103 (85%)	0 (0%)	**<0.001**
Significant fibrosis, n (%)	31 (21%)	31 (26%)	0 (0%)	**0.007**
Advanced fibrosis, n (%)	19 (13%)	19 (16%)	0 (0%)	**0.025**
Cirrhosis, n (%)	8 (5%)	8 (7%)	0 (0%)	0.352
**Glucose-lowering medications**				
Metformin, n (%)	111 (75%)	94 (78%)	17 (63%)	0.176
DPP-4 inhibitors, n (%)	16 (11%)	12 (10%)	4 (15%)	0. 494
GLP-1 RA, n (%)	16 (11%)	16 (13%)	0 (0%)	**0.044**
SGLT2 inhibitors, n (%)	7 (5%)	6 (5%)	1 (4%)	0.739
Thiazolidinediones, n (%)	4 (3%)	3 (2%)	1 (4%)	0.557
Sulfonylureas, n (%)	21 (14%)	17 (14%)	4 (15%)	1
Insulin, n (%)	18 (12%)	14 (12%)	4 (15%)	0.744
**Lipid-lowering medications**				
Statins, n (%)	104 (70%)	86 (71%)	18 (67%)	0.826
Fibrates, n (%)	7 (5%)	5 (4%)	2 (7%)	0.612
**Genetics**				
PNPLA3 rs738409 C>G*, n (%)				0.128
CC	54 (48%)	45 (48%)	9 (45%)	
CG	46 (41%)	35 (38%)	11 (55%)	
GG	13 (12%)	13 (14%)	0 (0%)	

(*continued on next page*)

Table 1. (*continued*)

	Overall	MASLD	Non-MASLD	*p* value
TM6SF2 rs58542926 C>T*, n (%)				**0.037**
CC	101 (89%)	86 (92%)	15 (75%)	
CT	12 (11%)	7 (8%)	5 (25%)	
TT	0 (0%)	0 (0%)	0 (0%)	
SERPINA1 rs28929474 C>T*, n (%)				0.547
CC	109 (96%)	90 (97%)	19 (95%)	
CT	4 (4%)	3 (3%)	1 (5%)	
TT	0 (0%)	0 (0%)	0 (0%)	
HSD17B13 rs72613567 T>TA*, n (%)				0.071
T/T	70 (62%)	53 (57%)	17 (85%)	
T/TA	40 (35%)	37 (40%)	3 (15%)	
TA/TA	3 (3%)	3 (3%)	0 (0%)	
PRS[#]	0 (0-1)	0 (0-1)	1 (0-1)	0.114
High PRS[#]	55 (49%)	43 (46%)	12 (60%)	0.384

Data are shown as mean (SD) or median (IQR) for continuous variables and frequencies (percentages) for categorical variables, as appropriate. *p* values are calculated using ANOVA, Kruskal-Wallis test, Chi-squared test, or Fisher's exact test as appropriate. Bold values denote statistical significance at *p* values <0.05.
Protection from MASLD defined as individuals not having steatosis (MRI-PDFF <5%) and significant fibrosis (MRE <3 kPa) at both baseline and 2-year follow-up visits. Steatosis defined as MRI-PDFF ≥5%. Significant fibrosis defined as MRE ≥3 kPa; advanced fibrosis defined as MRE ≥3.63 kPa; cirrhosis defined as MRE ≥4.67 kPa.
ALT, alanine aminotransferase; AST, aspartate aminotransferase; DPP-4, dipeptidyl peptidase 4; HOMA-IR, homeostasis model assessment of insulin resistance; GGT, gamma-glutamyltransferase; GLP-1 RA, glucagon-like peptide-1 receptor agonists; MASLD, metabolic dysfunction-associated steatotic liver disease; MRE, magnetic resonance elastography; MRI-PDFF, magnetic resonance imaging-derived proton density fat fraction; PRS, polygenic risk score; SGLT2, sodium-glucose cotransporter 2.
*Genotype distribution is in Hardy-Weinberg equilibrium.
[#]Unweighted PRS was calculated as the sum of established risk alleles in *PNPLA3*, *TM6SF2* and *SERPINA1* minus the protective variant in *HSD17B13*. PRS was dichotomized into high and low groups according to the median.

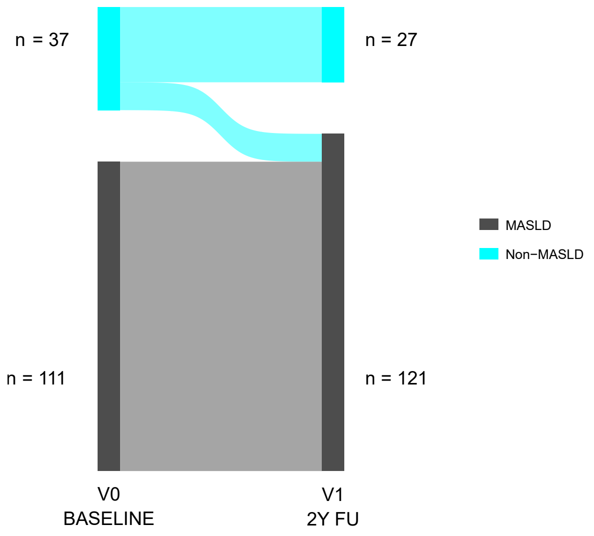

Fig. 1. Natural history of liver disease in individuals with type 2 diabetes and paired MRI/MRE assessments at baseline and 2-year follow-up (N = 148). Protection from liver disease development defined as individuals not having steatosis (MRI-PDFF <5%) and significant fibrosis (MRE <3 kPa) at both time points. MASLD, metabolic dysfunction-associated steatotic liver disease; MRE, magnetic resonance elastography; MRI-PDFF, magnetic resonance imaging-derived proton density fat fraction.

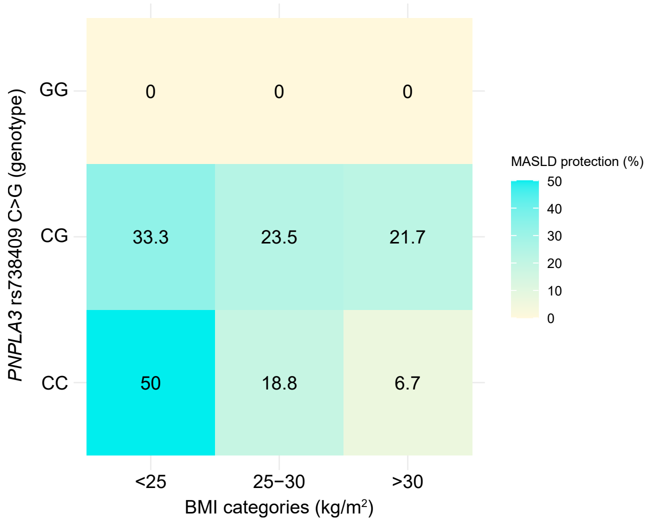

Fig. 2. Prevalence of protection from MASLD across BMI categories, stratified by *PNPLA3* rs738409 C>G genotype. Each box indicates the percentage of individuals with MASLD protection within the corresponding BMI × *PNPLA3* genotype subgroup. MASLD, metabolic dysfunction-associated steatotic liver disease.

lower circulating triglycerides were consistently identified as key predictors, supporting the robustness of these associations (Table S1, Figs. S3 and 4). Sex, HDL cholesterol, and the *PNPLA3* genotype were selected in all regularized models as predictors of protection from MASLD, although these factors did not reach statistical significance in the Firth's penalized model. Sensitivity analyses considering *PNPLA3* under a recessive model and including the PRS, type 2 diabetes duration, and statin therapy as covariates yielded results substantially similar to the main analysis (Tables S2–4).

Hepatic DNL in individuals with type 2 diabetes

To investigate potential biological mechanisms underlying a non-MASLD status in type 2 diabetes, we performed an exploratory analysis of hepatic DNL rates, measured via stable isotope tracing, in 11 individuals with type 2 diabetes from an independent cohort who similarly underwent liver MRI and MRE assessment. After 21 days of deuterium water labeling, mean DNL was significantly lower in individuals without hepatic steatosis, defined as MRI-PDFF <5%, compared to those with hepatic steatosis (25% *vs*. 37%, respectively; *p* = 0.032)

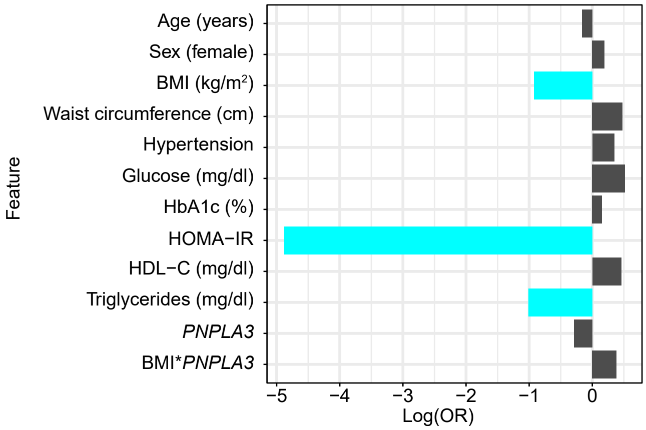

Fig. 3. Factors associated with protection from MASLD in individuals with type 2 diabetes. Log(OR) values were estimated using Firth logistic regression, with protection from liver disease development as the dependent variable. The bar plot displays the strength and direction of the association between each trait and the dependent variable. Positive log(OR) values indicate a direct association, whereas negative values indicate an inverse association. The magnitude of the log(OR) reflects the strength of the relationship. Predictors shown in blue were statistically significant in the model (p <0.05). For continuous predictors, effect sizes are presented as log(OR) for standardized variables (z-scores) to allow comparison across predictors. The corresponding ORs and confidence intervals from the unstandardized model are reported in the text. The *PNPLA3* rs738409 C>G was modeled additively, with effect per copy of the G allele. HOMA-IR, homeostasis model assessment of insulin resistance; log(OR), log odds ratios; MASLD, metabolic dysfunction-associated steatotic liver disease.

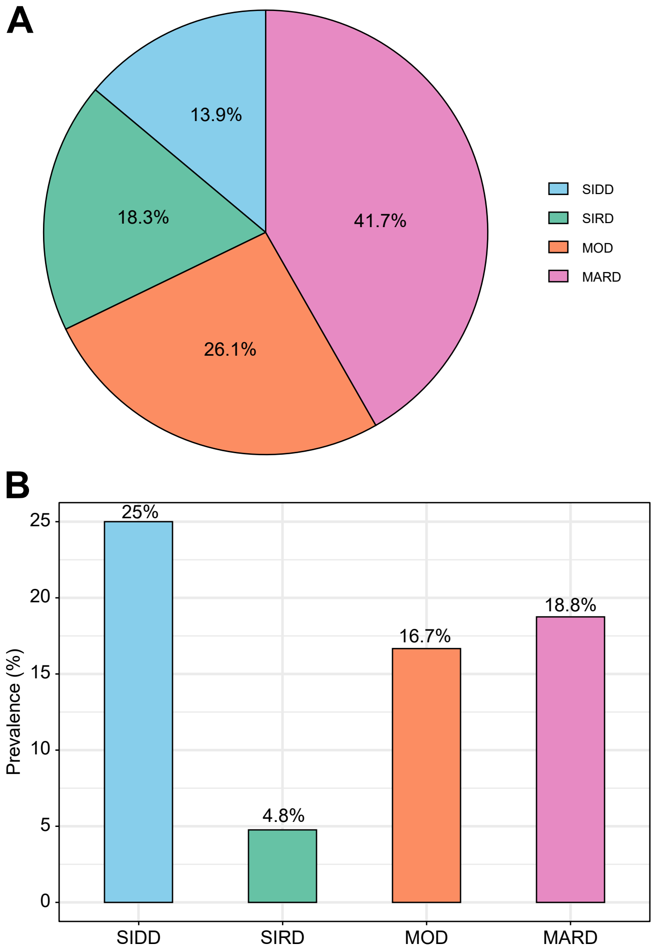

Fig. 4. Protection from MASLD across machine-learning–derived subtypes of type 2 diabetes. (A) Distribution of type 2 diabetes subtypes in the cohort with available data on diabetes duration (n = 115). (B) Prevalence of protection from MASLD stratified by type 2 diabetes subtype. MARD, mild age-related diabetes; MASLD, metabolic dysfunction-associated steatotic liver disease; MOD, mild obesity-related diabetes; SIDD, severe insulin-deficient diabetes; SIRD, severe insulin-resistant diabetes.

(Fig. 5). All individuals without hepatic steatosis also showed no evidence of significant fibrosis, defined as MRE <3 kPa.

Discussion

Using a uniquely characterized prospective cohort with longitudinal liver imaging using advanced MRI/MRE methods, we demonstrate that lower BMI, lower HOMA-IR, and lower circulating triglycerides are the strongest factors associated with protection from MASLD in type 2 diabetes. To our knowledge, the concept of factors conferring protection is novel and has not been systematically investigated, as prior research has predominantly focused on risk factors for liver disease development rather than on determinants supporting sustained resistance. Additionally, leveraging cutting-edge stable isotope tracing, we demonstrate that reduced hepatic DNL may represent a potential underlying biological mechanism contributing to a non-MASLD status in this population.

First, we used Firth's penalized logistic regression as the primary modeling approach to assess predictors associated with protection from MASLD. This method was selected due to its robustness in small samples and its ability to reduce bias in maximum likelihood estimates, particularly when events are rare, or separation occurs.[29,30] As complementary analyses, we employed a combination of regularized logistic regression models to enhance the predictive accuracy and robustness of our models.[31] Together, these approaches aim to mitigate overfitting and improve the reliability of parameter estimates in the context of complex, high-dimensional datasets. We identified lower BMI, lower HOMA-IR, and lower triglyceride levels as the strongest factors associated with protection from MASLD in individuals with type 2 diabetes, since they were consistently selected across all the logistic regression models. These results have important clinical implications, suggesting that managing modifiable risk factors, such as body weight and lipid profile, may be critical for preventing MASLD development and progression in this high-risk population.

Notably, sex, HDL cholesterol, and the *PNPLA3* genotype were selected as relevant predictors in all regularized logistic regression models, but they did not reach statistical significance in the Firth's penalized model. This suggests that these factors may also play a relevant role as predictors of protection from MASLD, warranting further investigation in larger longitudinal cohorts. Conversely, the duration of type 2 diabetes was not associated with protection from MASLD. This finding is consistent with a previous longitudinal analysis of over

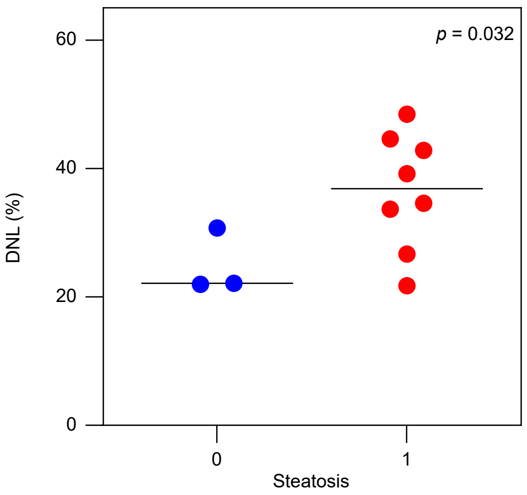

Fig. 5. Hepatic *de novo* lipogenesis in individuals with type 2 diabetes stratified by the presence of hepatic steatosis (MRI-PDFF ≥5%). *p* value calculated using unpaired t-test. DNL, *de novo* lipogenesis; MRI-PDFF, MRI-derived proton density fat fraction.

20,000 individuals with type 2 diabetes in the UK Biobank, in which disease duration was not linked to incident severe liver disease.[8]

The absence of homozygosity for the *PNPLA3* risk allele (GG) among protected individuals is consistent with a recessive effect of the G allele, aligning with its well-established role as a genetic risk factor.[32,33] However, the *PNPLA3* variant did not reach statistical significance in most multivariable models assessing protection from liver disease. This is likely attributable to the low frequency of the GG genotype in our cohort and the limited number of protected individuals. These factors contributed to wide confidence intervals and reduced statistical power, rather than indicating a true lack of association. Additionally, approximately 30% of our cohort were of Hispanic ethnicity, a group known to have a higher prevalence of the *PNPLA3* risk allele, predominantly in the heterozygous (CG) state. This may have diluted the observable effect in additive or dominant models. Lastly, given the well-established synergistic interaction between the *PNPLA3* I148M variant and BMI in MASLD,[34,35] the potential effect of *PNPLA3* on protection from MASLD may have been obscured by the stronger contribution of BMI in the multivariable model. Importantly, this further supports the notion that MASLD-associated polymorphisms exert minimal influence on disease onset and progression in the absence of obesity.[35]

Importantly, the distribution of protection across machine learning–derived type 2 diabetes subtypes revealed that individuals with the SIRD subtype, *i.e.* the most insulin-resistant group, exhibited the lowest prevalence of protection from MASLD. This pattern underscores the prominent contribution of insulin resistance to liver disease susceptibility in individuals with type 2 diabetes and aligns with our findings, in which lower HOMA-IR emerged as one of the strongest predictors of protection, as well as with previously published data reporting a higher risk of liver-related complications in the SIRD subgroup,[25–27] potentially informing precision medicine strategies.

Finally, we tested whether a non-MASLD status was related to reduced hepatic DNL, measured via stable isotope tracing. Previous studies using isotopes have shown that hepatic DNL is elevated in the context of insulin resistance and is associated with MASLD/MASH.[36–38] Our current findings demonstrate that lower hepatic DNL is associated with the absence of MASLD in individuals with type 2 diabetes. Importantly, our findings support the notion that increased hepatic DNL may represent the primary operative mechanism driving MASLD with a predominant metabolic component characterized by insulin resistance, hyperglycemia, and hyperinsulinemia.[2] Despite the limited sample size, which restricts statistical power and generalizability, this exploratory and hypothesis-generating cross-sectional analysis provides a biological basis for the clinical observations derived from our well-characterized prospective cohort and highlights hepatic DNL as a potentially targetable pathway for liver disease prevention in type 2 diabetes. Further longitudinal functional studies are needed to confirm these findings and to establish causality between reduced hepatic DNL and protection from MASLD in individuals with type 2 diabetes.

A major strength of this study lies in its well-characterized, diverse cohort of prospectively recruited participants who underwent comprehensive clinical assessments, including detailed liver phenotyping with advanced imaging techniques at both baseline and 2-year follow-up visits. Additionally, we leveraged stable isotope tracing with deuterium water to quantify hepatic DNL *in vivo*, one of the most precise and state-of-the-art methodologies for assessing metabolic dysfunction at a mechanistic level.

The main limitation of this study is the relatively small number of individuals exhibiting protection combined with the ethnic heterogeneity of the cohort, which may limit the ability to identify additional protective factors, including lower inherited risk related to *PNPLA3*, and the generalizability of findings. However, given the high prevalence of liver disease among individuals with type 2 diabetes, the population without liver disease represents a small subset of all individuals with type 2 diabetes. Furthermore, the use of MRI to detect liver fat ensures high diagnostic accuracy for the population resistant to liver disease. Additionally, the 2-year follow-up period may be insufficient to fully capture the long-term dynamics of MASLD progression or protection. Further longitudinal studies are needed to validate these findings and to assess the causal relationship between the identified factors and protection from MASLD.

In conclusion, in this well-characterized prospective cohort of individuals with type 2 diabetes, the strongest factors associated with protection from MASLD development were lower BMI, lower HOMA-IR, and lower triglycerides. Lower hepatic DNL may reflect a key underlying biological

mechanism. Our findings could help inform strategies to reduce the risk of liver disease progression in this high-risk population. They also contribute to clarifying the heterogeneity underlying MASLD pathogenesis and support the development of precision medicine strategies for its management.

Affiliations

[1]*MASLD Research Center, Division of Gastroenterology and Hepatology, University of California at San Diego, La Jolla, California, United States;* [2]*Departamento de Gastroenterología, Escuela de Medicina, Pontificia Universidad Católica de Chile, Santiago, Chile;* [3]*Department of Nutritional Sciences & Toxicology, University of California Berkeley, Berkeley, California, United States;* [4]*School of Public Health, University of California at San Diego, La Jolla, California, United States*

Abbreviations

DNL, *de novo* lipogenesis; HOMA-IR, homeostasis model assessment of insulin resistance; MASLD, metabolic dysfunction-associated steatotic liver disease; MRE, magnetic resonance elastography; MRI-PDFF, magnetic resonance imaging-derived proton density fat fraction; PRS, polygenic risk score.

Financial support

VA is supported by NIDDK (K23DK119460). RL receives funding support from NCATS (5UL1TR001442), NIDDK (U01DK061734, U01DK130190, R01DK106419, R01DK121378, R01DK124318, P30DK120515), NHLBI (P01HL147835), John C Martin Foundation (RP124).

Conflicts of interest

RL serves as a consultant to Aardvark Therapeutics, Altimmune, Arrowhead Pharmaceuticals, AstraZeneca, Cascade Pharmaceuticals, Eli Lilly, Gilead, Glympse bio, Inipharma, Intercept, Inventiva, Ionis, Janssen Inc., Lipidio, Madrigal, Neurobo, Novo Nordisk, Merck, Pfizer, Sagimet, 89 bio, Takeda, Terns Pharmaceuticals and Viking Therapeutics. In addition, his institution received research grants from Arrowhead Pharmaceuticals, Astrazeneca, Boehringer-Ingelheim, Bristol-Myers Squibb, Eli Lilly, Galectin Therapeutics, Gilead, Intercept, Hanmi, Intercept, Inventiva, Ionis, Janssen, Madrigal Pharmaceuticals, Merck, Novo Nordisk, Pfizer, Sonic Incytes and Terns Pharmaceuticals. Co-founder of LipoNexus Inc.

Please refer to the accompanying ICMJE disclosure forms for further details.

Authors' contributions

FT and RL contributed to study concept and design. FT and RL contributed to drafting the manuscript. FT contributed to perform the statistical analysis. All authors contributed to the analysis and interpretation of data, critically revised the manuscript for important intellectual content, and approved the final version of the manuscript. RL is the guarantor of this work and, as such, had full access to all the data in the study and takes responsibility for the integrity of the data and the accuracy of the data analysis.

Data availability

All data supporting the findings of this study are available within the article.

Acknowledgements

The authors thank the staff and the participants of the DOS study.

Supplementary data

Supplementary data to this article can be found online at https://doi.org/10.1016/j.jhepr.2026.101733.

References

Author names in bold designate shared co-first authorship

[1] Targher G, Valenti L, Byrne CD. Metabolic dysfunction-associated steatotic liver disease. N Engl J Med 2025;393:683–698.

[2] Stefan N, Yki-Järvinen H, Neuschwander-Tetri BA. Metabolic dysfunction-associated steatotic liver disease: heterogeneous pathomechanisms and effectiveness of metabolism-based treatment. Lancet Diabetes Endocrinol 2025;13:134–148.

[3] Byrne CD, Armandi A, Pellegrinelli V, et al. Metabolic dysfunction-associated steatotic liver disease: a condition of heterogeneous metabolic risk factors, mechanisms and comorbidities requiring holistic treatment. Nat Rev Gastroenterol Hepatol 2025;22:314–328.

[4] **Raverdy V**, **Tavaglione F**, **Chatelain E**, et al. Performance of non-invasive tests for liver fibrosis resolution after bariatric surgery. Metabolism 2024;153:155790.

[5] **Raverdy V**, **Tavaglione F**, **Chatelain E**, et al. Data-driven cluster analysis identifies distinct types of metabolic dysfunction-associated steatotic liver disease. Nat Med 2024;30:3624–3633.

[6] Jamialahmadi O, De Vincentis A, Tavaglione F, et al. Partitioned polygenic risk scores identify distinct types of metabolic dysfunction-associated steatotic liver disease. Nat Med 2024;30:3614–3623.

[7] Huang DQ, Wilson LA, Behling C, et al. Liver stiffness progression in biopsy-proven metabolic dysfunction-associated steatotic disease among people with diabetes versus people without diabetes: a prospective multicenter study. Hepatology 2025;81:1553–1563.

[8] **Tavaglione F**, **De Vincentis A**, Jamialahmadi O, et al. Inborn and acquired risk factors for severe liver disease in Europeans with type 2 diabetes from the UK Biobank. JHEP Rep 2021;3:100262.

[9] Huang DQ, Wilson LA, Behling C, et al. Fibrosis progression rate in biopsy-proven nonalcoholic fatty liver disease among people with diabetes versus people without diabetes: a multicenter study. Gastroenterology 2023;165:463–472.e5.

[10] En Li Cho E, Ang CZ, Quek J, et al. Global prevalence of non-alcoholic fatty liver disease in type 2 diabetes mellitus: an updated systematic review and meta-analysis. Gut 2023;72:2138–2148.

[11] Ajmera V, Cepin S, Tesfai K, et al. A prospective study on the prevalence of NAFLD, advanced fibrosis, cirrhosis and hepatocellular carcinoma in people with type 2 diabetes. J Hepatol 2023;78:471–478.

[12] Huang DQ, Noureddin N, Ajmera V, et al. Type 2 diabetes, hepatic decompensation, and hepatocellular carcinoma in patients with non-alcoholic fatty liver disease: an individual participant-level data meta-analysis. Lancet Gastroenterol Hepatol 2023;8:829–836.

[13] Cuschieri S. The STROBE guidelines. Saudi J Anaesth 2019;13:S31–S34.

[14] Caussy C, Alquiraish MH, Nguyen P, et al. Optimal threshold of controlled attenuation parameter with MRI-PDFF as the gold standard for the detection of hepatic steatosis. Hepatology 2018;67:1348–1359.

[15] Amangurbanova M, Huang DQ, Noureddin N, et al. A prospective study on the prevalence of MASLD in patients with type 2 diabetes and hyperferritinaemia. Aliment Pharmacol Ther 2025;61:456–464.

[16] **Hsu C**, **Caussy C**, Imajo K, et al. Magnetic resonance vs transient elastography analysis of patients with nonalcoholic fatty liver disease: a systematic review and pooled analysis of individual participants. Clin Gastroenterol Hepatol 2019;17:630–637.e8.

[17] **Romeo S**, **Kozlitina J**, Xing C, et al. Genetic variation in PNPLA3 confers susceptibility to nonalcoholic fatty liver disease. Nat Genet 2008;40:1461–1465.

[18] **Kozlitina J**, **Smagris E**, Stender S, et al. Exome-wide association study identifies a TM6SF2 variant that confers susceptibility to nonalcoholic fatty liver disease. Nat Genet 2014;46:352–356.

[19] **Jermy B**, **Brownrigg J**, Strnad P, et al. Alpha-1 antitrypsin Pi*MZ variant increases the risk of liver disease progression in MASLD and MASH. Ann Hepatol 2025:102143.

[20] Abul-Husn NS, Cheng X, Li AH, et al. A protein-truncating HSD17B13 variant and protection from chronic liver disease. N Engl J Med 2018;378:1096–1106.

[21] Bridi L, Agrawal S, Tesfai K, et al. The impact of genetic risk on the prevalence of advanced fibrosis and cirrhosis in prospectively assessed patients with type 2 diabetes. Aliment Pharmacol Ther 2024;60:369–377.

[22] Hellerstein MK, Neese RA. Mass isotopomer distribution analysis: a technique for measuring biosynthesis and turnover of polymers. Am J Physiol 1992;263:E988–E1001.

[23] Rinella ME, Lazarus JV, Ratziu V, et al. A multi-society Delphi consensus statement on new fatty liver disease nomenclature. Hepatology 2023;78:1966–1986.

[24] Ahlqvist E, Storm P, Käräjämäki A, et al. Novel subgroups of adult-onset diabetes and their association with outcomes: a data-driven cluster analysis of six variables. Lancet Diabetes Endocrinol 2018;6:361–369.
[25] Zaharia OP, Strassburger K, Strom A, et al. Risk of diabetes-associated diseases in subgroups of patients with recent-onset diabetes: a 5-year follow-up study. Lancet Diabetes Endocrinol 2019;7:684–694.
[26] **Otero Sanchez L**, **Zhan CY**, Gomes da Silveira Cauduro C, et al. A machine learning-based classification of adult-onset diabetes identifies patients at risk of liver-related complications. JHEP Rep 2023;5:100791.
[27] Asplund O, Thangam M, Prasad RB, et al. Comorbidities and mortality in subgroups of adults with diabetes with up to 14 years follow-up: a prospective cohort study in Sweden. Lancet Diabetes Endocrinol 2026;14:29–40.
[28] Wallace TM, Levy JC, Matthews DR. Use and abuse of HOMA modeling. Diabetes Care 2004;27:1487–1495.
[29] Firth D. Bias reduction of maximum likelihood estimates. Biometrika 1993;80:27–38.
[30] Suhas S, Manjunatha N, Kumar CN, et al. Firth's penalized logistic regression: a superior approach for analysis of data from India's National Mental Health Survey, 2016. Indian J Psychiatry 2023;65:1208–1213.
[31] **Jamialahmadi O**, **Tavaglione F**, Rawshani A, et al. Fatty liver disease, heart rate and cardiac remodelling: evidence from the UK Biobank. Liver Int 2023;43:1247–1255.
[32] Díaz LA, Alazawi W, Agrawal S, et al. High inherited risk predicts age-associated increases in fibrosis in patients with MASLD. J Hepatol 2025;83:849–859.
[33] **De Vincentis A**, **Tavaglione F**, Jamialahmadi O, et al. A polygenic risk score to refine risk stratification and prediction for severe liver disease by clinical fibrosis scores. Clin Gastroenterol Hepatol 2022;20:658–673.
[34] Stender S, Kozlitina J, Nordestgaard BG, et al. Adiposity amplifies the genetic risk of fatty liver disease conferred by multiple loci. Nat Genet 2017;49:842–847.
[35] Mann JP, Anstee QM. NAFLD: PNPLA3 and obesity: a synergistic relationship in NAFLD. Nat Rev Gastroenterol Hepatol 2017;14:506–507.
[36] Lambert JE, Ramos-Roman MA, Browning JD, et al. Increased de novo lipogenesis is a distinct characteristic of individuals with nonalcoholic fatty liver disease. Gastroenterology 2014;146:726–735.
[37] **Lawitz EJ**, **Li KW**, Nyangau E, et al. Elevated de novo lipogenesis, slow liver triglyceride turnover, and clinical correlations in nonalcoholic steatohepatitis patients. J Lipid Res 2022;63:100250.
[38] Smith GI, Shankaran M, Yoshino M, et al. Insulin resistance drives hepatic de novo lipogenesis in nonalcoholic fatty liver disease. J Clin Invest 2020;130:1453–1460.

Keywords: NAFLD/MASLD; insulin resistance; de novo lipogenesis; body mass index; triglycerides.

Received 20 October 2025; received in revised form 5 December 2025; accepted 22 December 2025; Available online 8 January 2026

Journal of Hepatology, Volume *8*

Supplemental information

Factors associated with protection from MASLD in type 2 diabetes: A prospective study integrating longitudinal MRI/MRE and stable isotope tracing

Federica Tavaglione, Veeral Ajmera, Luis Antonio Díaz, Kelvin Li, Egbert Madamba, Ricki Bettencourt, Lisa Richards, Marc Hellerstein, and Rohit Loomba

SUPPLEMENTARY MATERIAL

Factors associated with protection from MASLD in type 2 diabetes: A prospective study integrating longitudinal MRI/MRE and stable isotope tracing

Federica Tavaglione[1], Veeral Ajmera[1], Luis Antonio Díaz[1,2], Kelvin Li[3], Egbert Madamba[1], Ricki Bettencourt[1], Lisa Richards[1], Marc Hellerstein[3], Rohit Loomba[1,4]

[1] MASLD Research Center, Division of Gastroenterology and Hepatology, University of California at San Diego, La Jolla, California, United States.
[2] Departamento de Gastroenterología, Escuela de Medicina, Pontificia Universidad Católica de Chile, Santiago, Chile.
[3] Department of Nutritional Sciences & Toxicology, University of California Berkeley, Berkeley, California, United States.
[4] School of Public Health, University of California at San Diego, La Jolla, California, United States.

Table of Contents

SUPPLEMENTARY TABLE 1: FACTORS ASSOCIATED WITH PROTECTION FROM MASLD IN INDIVIDUALS WITH TYPE 2 DIABETES. 3

SUPPLEMENTARY TABLE 2: FACTORS ASSOCIATED WITH PROTECTION FROM MASLD IN INDIVIDUALS WITH TYPE 2 DIABETES, CONSIDERING *PNPLA3* UNDER A RECESSIVE MODEL. 4

SUPPLEMENTARY TABLE 3: FACTORS ASSOCIATED WITH PROTECTION FROM MASLD IN INDIVIDUALS WITH TYPE 2 DIABETES, INCLUDING THE POLYGENIC RISK SCORE (PRS) AS A COVARIATE. 5

SUPPLEMENTARY TABLE 4: FACTORS ASSOCIATED WITH PROTECTION FROM MASLD IN INDIVIDUALS WITH TYPE 2 DIABETES, INCLUDING STATIN THERAPY AND TYPE 2 DIABETES DURATION AS COVARIATES. 6

SUPPLEMENTARY FIGURE 1: STUDY FLOWCHART. 7

SUPPLEMENTARY FIGURE 2: COMPARISON OF ORIGINAL AND IMPUTED DISTRIBUTIONS USING DENSITY PLOTS FOR CONTINUOUS VARIABLES AND BAR PLOTS FOR THE CATEGORICAL VARIABLES 8

SUPPLEMENTARY FIGURE 3: FACTORS ASSOCIATED WITH PROTECTION FROM MASLD IN INDIVIDUALS WITH TYPE 2 DIABETES USING REGULARIZED LOGISTIC REGRESSION MODELS. 9

SUPPLEMENTARY FIGURE 4: OVERLAP OF PREDICTORS SELECTED BY BIAS-REDUCED AND PENALIZED LOGISTIC REGRESSION MODELS. 10

Supplementary Table 1: Factors associated with protection from MASLD in individuals with type 2 diabetes.

	**OR (95%CI)**	**p-value**
Age (years)	0.98 (0.90, 1.06)	0.594
Sex (Female)	1.21 (0.35, 4.39)	0.758
BMI (kg/m^2)	0.81 (0.64, 0.98)	**0.029**
Waist circumference (cm)	1.04 (0.98, 1.11)	0.178
Hypertension	1.41 (0.51, 4.13)	0.509
Glucose (mg/dL)	1.01 (0.99, 1.03)	0.274
HbA1c (%)	1.13 (0.51, 2.56)	0.757
HOMA-IR	0.59 (0.39, 0.83)	**8.6e-4**
HDL cholesterol (mg/dL)	1.04 (0.99, 1.08)	0.103
Triglycerides (mg/dL)	0.986 (0.973, 0.997)	**0.011**
PNPLA3	0.05 (7.5e-5, 14.40)	0.303
BMI**PNPLA3*	1.09 (0.90, 1.36)	0.380

Outcome: MASLD protection defined as individuals not having steatosis (MRI-PDFF < 5%) and significant fibrosis (MRE < 3 kPa) at both baseline and 2-year follow-up visits.

ORs > 1 indicate increased odds of protection (i.e., higher values of the feature are associated with a higher likelihood of being protected).

ORs < 1 indicate decreased odds of protection (i.e., higher values of the feature are associated with a lower likelihood of being protected).

P values are calculated using Firth logistic regression. Bold values denote statistical significance at p-value <0.05. The *PNPLA3* rs738409 C>G was used as additive model.

Abbreviations: BMI, body mass index; CI, confidence interval; HbA1c, hemoglobin A1c; HOMA-IR, homeostasis model assessment of insulin resistance; OR, odds ratio; *PNPLA3*, patatin-like phospholipase domain-containing protein 3.

Supplementary Table 2: Factors associated with protection from MASLD in individuals with type 2 diabetes, considering *PNPLA3* under a recessive model.

	**OR (95%CI)**	**p-value**
Age (years)	0.97 (0.90, 1.04)	0.386
Sex (Female)	1.32 (0.38, 4.90)	0.663
BMI (kg/m^2)	0.83 (0.69, 0.98)	**0.031**
Waist circumference (cm)	1.04 (0.99, 1.11)	0.141
Hypertension	1.40 (0.49, 4.14)	0.530
Glucose (mg/dL)	1.01 (0.99, 1.04)	0.199
HbA1c (%)	0.99 (0.45, 2.21)	0.971
HOMA-IR	0.58 (0.38, 0.83)	**1.10e-3**
HDL cholesterol (mg/dL)	1.03 (0.99, 1.08)	0.189
Triglycerides (mg/dL)	0.986 (0.973, 0.997)	**0.008**
PNPLA3	0.017 (1.21e-21, 4.79e7)	0.629
BMI**PNPLA3*	1.08 (0.44, 4.22)	0.798

Outcome: MASLD protection defined as individuals not having steatosis (MRI-PDFF < 5%) and significant fibrosis (MRE < 3 kPa) at both baseline and 2-year follow-up visits.

ORs > 1 indicate increased odds of protection (i.e., higher values of the feature are associated with a higher likelihood of being protected).

ORs < 1 indicate decreased odds of protection (i.e., higher values of the feature are associated with a lower likelihood of being protected).

P values are calculated using Firth logistic regression. Bold values denote statistical significance at p-value <0.05. The *PNPLA3* rs738409 C>G was used as recessive model.

Abbreviations: BMI, body mass index; CI, confidence interval; HbA1c, hemoglobin A1c; HOMA-IR, homeostasis model assessment of insulin resistance; OR, odds ratio; *PNPLA3*, patatin-like phospholipase domain-containing protein 3.

Supplementary Table 3: Factors associated with protection from MASLD in individuals with type 2 diabetes, including the polygenic risk score (PRS) as a covariate.

	**OR (95%CI)**	**p-value**
Age (years)	0.997 (0.92, 1.09)	0.945
Sex (Female)	1.85 (0.44, 8.68)	0.405
BMI (kg/m^2)	0.82 (0.65, 0.999)	0.**048**
Waist circumference (cm)	1.06 (1.00, 1.14)	0.064
Hypertension	1.53 (0.48, 5.22)	0.469
Glucose (mg/dL)	1.02 (1.00, 1.05)	0.070
HbA1c (%)	0.87 (0.36, 2.12)	0.764
HOMA-IR	0.65 (0.43, 0.89)	**0.007**
HDL cholesterol (mg/dL)	1.01 (0.96, 1.06)	0.649
Triglycerides (mg/dL)	0.986 (0.969, 0.999)	**0.027**
PRS	1.01 (0.03, 45.99)	0.997
BMI*PRS	1.00 (0.88, 1.14)	0.944

Missing values for genetic variants were excluded from the analysis (n = 113).

Outcome: MASLD protection defined as individuals not having steatosis (MRI-PDFF < 5%) and significant fibrosis (MRE < 3 kPa) at both baseline and 2-year follow-up visits.

ORs > 1 indicate increased odds of protection (i.e., higher values of the feature are associated with a higher likelihood of being protected).

ORs < 1 indicate decreased odds of protection (i.e., higher values of the feature are associated with a lower likelihood of being protected).

P values are calculated using Firth logistic regression. Bold values denote statistical significance at p-value <0.05.

Unweighted PRS was calculated as the sum of established risk alleles in *PNPLA3*, *TM6SF2* and *SERPINA1* minus the protective variant in *HSD17B13*.

Abbreviations: BMI, body mass index; CI, confidence interval; HbA1c, hemoglobin A1c; HOMA-IR, homeostasis model assessment of insulin resistance; OR, odds ratio; PRS, polygenic risk score.

Supplementary Table 4: Factors associated with protection from MASLD in individuals with type 2 diabetes, including statin therapy and type 2 diabetes duration as covariates.

	OR (95%CI)	p-value	OR (95%CI)*	p-value
Age (years)	0.98 (0.91, 1.06)	0.637	0.98 (0.90, 1.08)	0.731
Sex (Female)	1.27 (0.37, 4.56)	0.709	1.83 (0.37, 11.04)	0.466
Diabetes duration (years)	-		1.08 (0.98, 1.20)	0.111
Statins (yes)	0.72 (0.24, 2.14)	0.545	0.70 (0.18, 2.85)	0.610
BMI (kg/m^2)	0.81 (0.65, 0.99)	**0.035**	0.87 (0.69, 1.07)	0.200
Waist circumference (cm)	1.04 (0.98, 1.10)	0.210	1.03 (0.95, 1.11)	0.488
Hypertension	1.37 (0.48, 4.01)	0.553	1.38 (0.40, 5.10)	0.609
Glucose (mg/dL)	1.01 (0.99, 1.03)	0.325	1.02 (0.99, 1.05)	0.215
HbA1c (%)	1.14 (0.51, 2.58)	0.750	0.74 (0.24, 2.17)	0.592
HOMA-IR	0.59 (0.39, 0.83)	**0.001**	0.63 (0.38, 0.90)	**0.007**
HDL cholesterol (mg/dL)	1.03 (0.99, 1.08)	0.119	1.04 (0.99, 1.09)	0.169
Triglycerides (mg/dL)	0.986 (0.973, 0.997)	**0.010**	0.985 (0.968, 0.998)	**0.024**
PNPLA3	0.06 (8.88e-05, 16.01)	0.324	0.02 (7.07e-06, 10.06)	0.220
BMI*PNPLA3*	1.09 (0.89, 1.36)	0.401	1.13 (0.91, 1.47)	0.273

* Missing values for type 2 diabetes duration were excluded from the analysis (n = 115).

Outcome: MASLD protection defined as individuals not having steatosis (MRI-PDFF < 5%) and significant fibrosis (MRE < 3 kPa) at both baseline and 2-year follow-up visits.

ORs > 1 indicate increased odds of protection (i.e., higher values of the feature are associated with a higher likelihood of being protected).

ORs < 1 indicate decreased odds of protection (i.e., higher values of the feature are associated with a lower likelihood of being protected).

P values are calculated using Firth logistic regression. Bold values denote statistical significance at p-value <0.05. The *PNPLA3* rs738409 C>G was used as additive model.

Abbreviations: BMI, body mass index; CI, confidence interval; HbA1c, hemoglobin A1c; HOMA-IR, homeostasis model assessment of insulin resistance; OR, odds ratio; *PNPLA3*, patatin-like phospholipase domain-containing protein 3.

Supplementary Figure 1: Study flowchart.

DOS study

N = 622 (MARCH 2025)

Inclusion criteria

- Age ≥ 50 years and <80 years
- Type 2 diabetes according to the ADA clinical practice recommendations
- Written informed consent

**Baseline**	622
**2-year follow-up**	204

Exclusion criteria

	**Baseline**	**2-year follow-up**
**MRI-PDFF NA**	56	34
**MRE NA**	95	37
**Alcohol >20/30 g/day (F/M)**	20	-
**Alcohol g/day NA**	32	-

n = 148

Inclusion criteria

- Paired MRI and MRE at baseline and 2-year follow-up

**Baseline**	148
**2-year follow-up**	148

Supplementary Figure 2: Comparison of original and imputed distributions using density plots for continuous variables and bar plots for the categorical variables. *Abbreviations*: HbA1c, hemoglobin A1c; HDL, high-density lipoprotein; HOMA-IR, homeostasis model assessment of insulin resistance; *PNPLA3*, patatin-like phospholipase domain-containing protein 3.

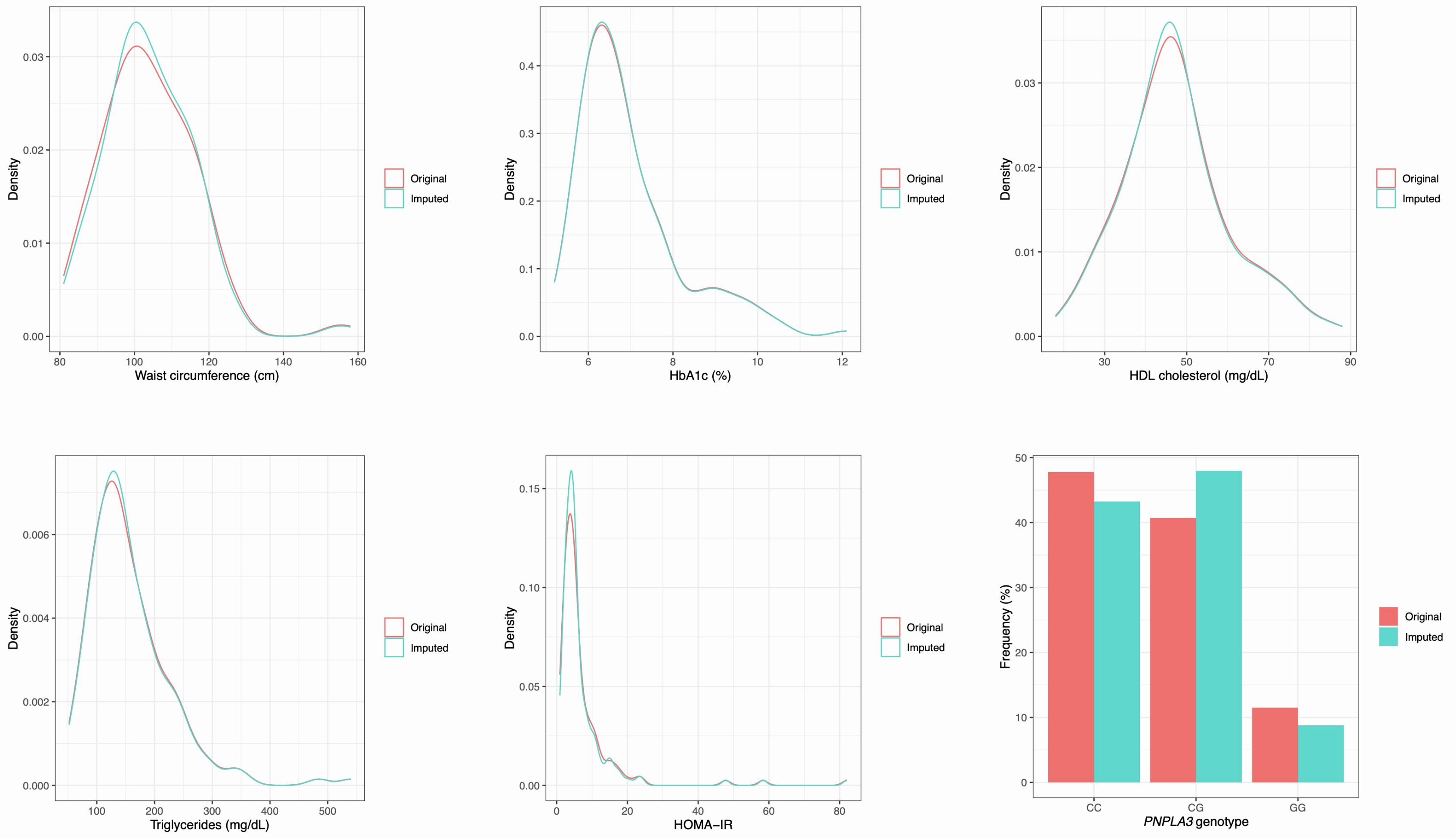

Supplementary Figure 3: Factors associated with protection from MASLD in individuals with type 2 diabetes using regularized logistic regression models. All continuous predictors were standardized (z-scores) prior to inclusion in the regularized regression models to ensure appropriate penalization and variable selection. *Abbreviations*: BMI, body mass index; HbA1c, hemoglobin A1c; HDL-C, high-density lipoprotein cholesterol; HOMA-IR, homeostasis model assessment of insulin resistance; *PNPLA3*, patatin-like phospholipase domain-containing protein 3.

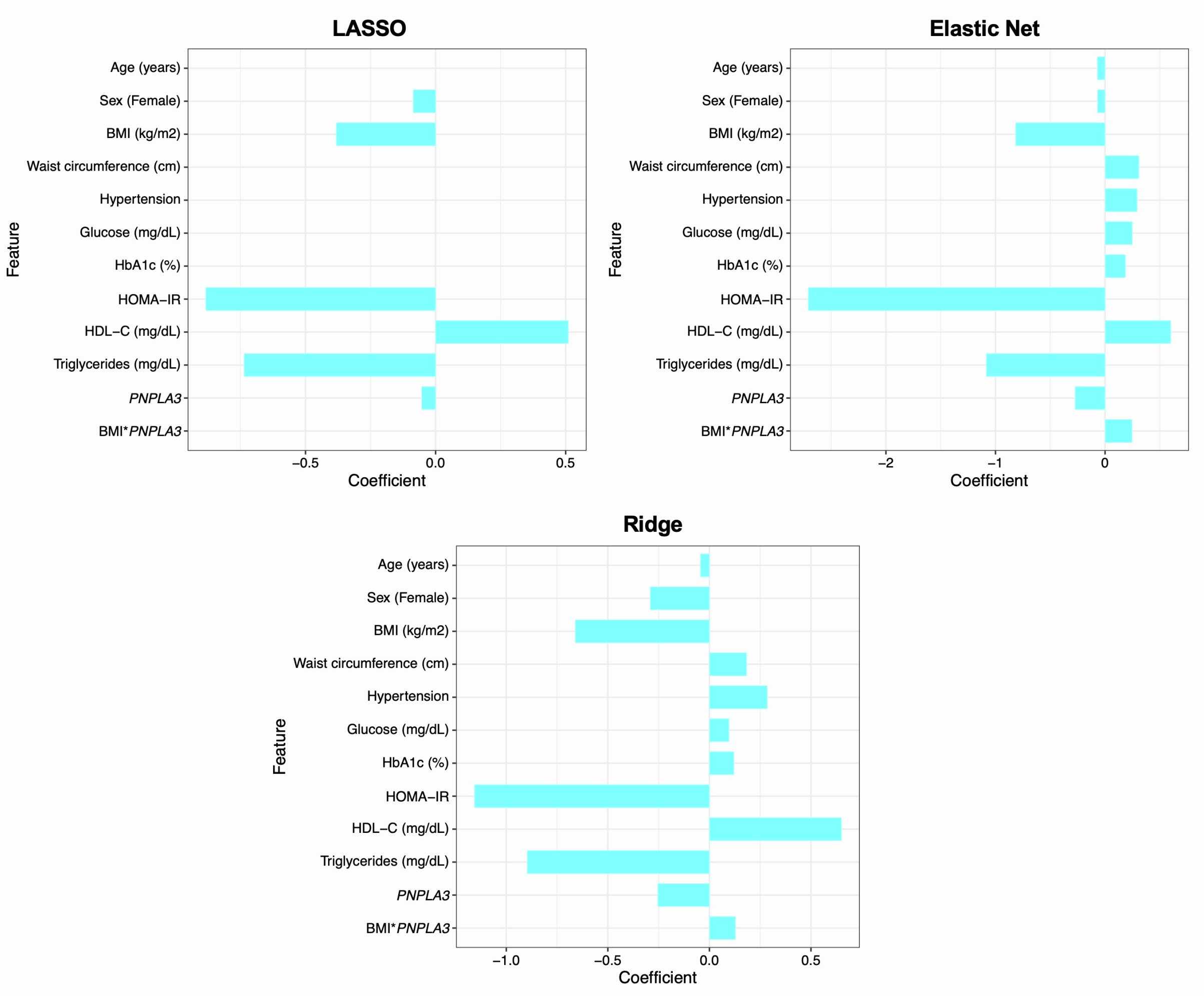

Supplementary Figure 4: Overlap of predictors selected by bias-reduced and penalized logistic regression models. The Euler diagram shows the overlap of predictors retained by Firth logistic regression, LASSO, Ridge, and Elastic Net models. Each method applies different regularization or bias reduction strategies to address multicollinearity and overfitting. Common predictors across models suggest robust associations with the outcome. Predictors consistently selected by all models are displayed in bold. *Abbreviations*: BMI, body mass index; HbA1c, hemoglobin A1c; HDL-C, high-density lipoprotein cholesterol; HOMA-IR, homeostasis model assessment of insulin resistance; *PNPLA3*, patatin-like phospholipase domain-containing protein 3.

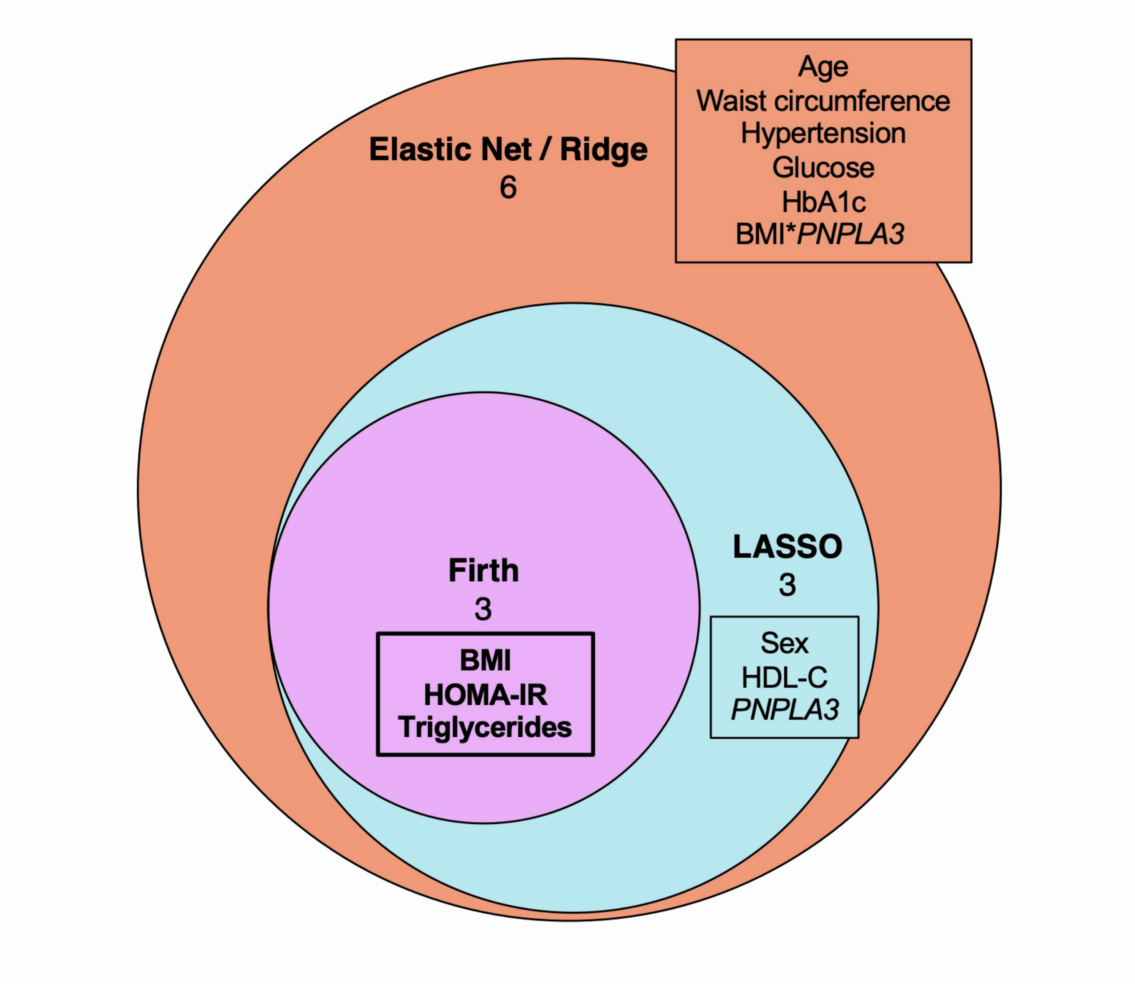

Supplement: Multimedia component 4 [file mmc4.pdf]
